# The Complex Transcriptional Response of *Acaryochloris marina* to Different Oxygen Levels

**DOI:** 10.1534/g3.116.036855

**Published:** 2016-12-14

**Authors:** Miguel A. Hernández-Prieto, Yuankui Lin, Min Chen

**Affiliations:** Australian Research Council Centre of Excellence for Translational Photosynthesis and School of Biological Sciences, University of Sydney, New South Wales 2006, Australia

**Keywords:** cyanobacteria, oxygen levels, transcriptome response, chlorophyll biosynthesis, reactive oxygen species

## Abstract

Ancient oxygenic photosynthetic prokaryotes produced oxygen as a waste product, but existed for a long time under an oxygen-free (anoxic) atmosphere, before an oxic atmosphere emerged. The change in oxygen levels in the atmosphere influenced the chemistry and structure of many enzymes that contained prosthetic groups that were inactivated by oxygen. In the genome of *Acaryochloris marina*, multiple gene copies exist for proteins that are normally encoded by a single gene copy in other cyanobacteria. Using high throughput RNA sequencing to profile transcriptome responses from cells grown under microoxic and hyperoxic conditions, we detected 8446 transcripts out of the 8462 annotated genes in the Cyanobase database. Two-thirds of the 50 most abundant transcripts are key proteins in photosynthesis. Microoxic conditions negatively affected the levels of expression of genes encoding photosynthetic complexes, with the exception of some subunits. In addition to the known regulation of the multiple copies of *psbA*, we detected a similar transcriptional pattern for *psbJ* and *psbU*, which might play a key role in the altered components of photosystem II. Furthermore, regulation of genes encoding proteins important for reactive oxygen species-scavenging is discussed at genome level, including, for the first time, specific small RNAs having possible regulatory roles under varying oxygen levels.

Photosynthesis uses solar energy, and transforms it into chemical energy, which is stored within the organic molecules of the organism. In essence, it provides the energy for all life on our planet. During oxygenic photosynthesis, sunlight is funneled toward a special pair of chlorophyll molecules, which produce a charge separation that results in the extraction of electrons from water. This initiates a chain of redox reactions that power the fixation of inorganic carbon into 3-phosphoglycerate, with oxygen (O_2_) generated as a side product of this reaction. In fact, before photosynthesis occurred in ancient cyanobacteria around 3.5–2.4 billion yr ago, the atmosphere was largely anaerobic (Blankenship and Hartman 1998). Over billions of years, oxygenic photosynthetic organisms changed the Earth’s atmosphere, steadily increasing its O_2_ levels to over 21% (v/v). This permitted the rise of multicellular organisms, dependent upon aerobic respiration (Dismukes *et al.* 2001; Blankenship 2008; Hedges *et al.* 2004).

Aerobic respiration is highly efficient in recovering the energy contained within the chemical bonds of organic molecules through oxidative phosphorylation. However, organisms living in aerobic environments also run the risk of being damaged by oxidants and reactive oxygen species (ROS). ROS include a number of reactive molecules derived from O_2_. Clearly, O_2_ in its ground state is harmless, as it has two unpaired electrons with parallel spin, making it paramagnetic. In this form, it is unlikely to participate in reactions with organic molecules, unless it is enzymatically or chemically activated by other reactions (Apel and Hirt 2004; Sharma *et al.* 2012). However, oxygen-derived ROS comprise superoxide, hydrogen peroxide, and hydroxyl radicals, which are a threat to the cell. Organisms mitigate ROS deleterious effects in various ways: by scavenging pathways (Ślesak *et al.* 2016; Pospíšil 2012), by changing the regulation of affected genes (Tamagnini *et al.* 2007), by separating the location of their product to oxygen-free compartments like heterocysts (Murry *et al.* 1984), or by evolving an alternative pathway resistant to oxidation (Busch and Montgomery 2015). Many metabolic pathways functioning today still contain enzymes sensitive to O_2_ levels, as illustrated by the coexisting oxygen-dependent, or oxygen-independent, reactions within the tetrapyrrole biosynthetic pathway (Busch and Montgomery 2015; Raymond and Blankenship 2004), and the activation of a counterpart D1 subunit of photosystem II (PSII) in response to changed O_2_ levels (Summerfield *et al.* 2008).

*Acaryochloris marina* (hereafter *Acaryochloris*) is a unicellular cyanobacterium, using chlorophyll *d* (Chl *d*), instead of chlorophyll *a* (Chl *a*), as the major pigment in its photosystems (Chen *et al.* 2002a, 2005b). Similar to all cyanobacteria, the thylakoids of *Acaryochloris* need to mitigate not only the oxidative stress generated by oxygenic photosynthetic activities, but also the oxidative stress produced because of aerobic respiration. Therefore, it is not surprising that under illumination, especially high-intensity light, singlet oxygen is mainly produced because of the interaction of unquenched Chl triplets with O_2_ generated within PSII, and the water-splitting complex. In photosystem I (PSI), the univalent reduction of O_2_ generates mainly superoxide anion radicals (reviewed in Latifi *et al.* 2009; Rutherford *et al.* 2012). To reduce the effects of ROS, a constant diffusion of O_2_ through the cell and photosynthetic membranes under illumination is crucial. This diffusion of O_2_, along with antioxidant enzymes that prevent accumulation of ROS, and the existence of remediation metabolites, such as ascorbic acid, glutathione, tocopherols, carotenoids, and flavonoids, prevent the interaction of O_2_ with electrons, other than those in the normal electron transfer pathways to O_2_, avoiding any possible oxidative stress (Latifi *et al.* 2009).

Because of the iconic character of *Acaryochloris*, in which the function of Chl *a* has been largely replaced by red-shifted Chl *d*, most of the research on this organism has been directed toward understanding Chl *d* biosynthesis (Schliep *et al.* 2010; Loughlin *et al.* 2014; Yoneda *et al.* 2016). In particular, this has focused on its role in photosynthesis (Chen *et al.* 2002b, 2005b; Hu *et al.* 1998; Tomo *et al.* 2007), and in far-red light acclimation (Duxbury *et al.* 2009). These studies have revealed direct oxidation of Chl *a* to Chl *d*, with participation of O_2_ (Schliep *et al.* 2010; Loughlin *et al.* 2013). Although the structural difference between Chl *a* and Chl *d* has a significant effect on its spectral characteristics, it does not affect the binding of Chl *d* with typical Chl *a* binding-peptides, as shown in *in vitro* reconstitution experiments (Chen *et al.* 2005a; Hoober *et al.* 2007; Chen and Cai 2007). In fact, so far, none of the studies carried out on the photosystems of *Acaryochloris* have revealed any significant difference to Chl *a*-containing photosystems, besides their distinct spectral characteristics, related to their pigment substitutions (Chen *et al.* 2005b).

In this study, we grew *Acaryochloris* under different O_2_ levels to test the effects of O_2_ on photo-pigment biosynthesis, photosynthetic reactions, and on their relationship with other essential metabolic reactions (including DNA and protein metabolism). Using high-throughput RNA sequencing (RNAseq), we obtained genome-level information on all expressed transcripts, under microoxic, normal air (control), and hyperoxic conditions. We detected genes coding for key proteins in photosynthesis and synthesis of chlorophyll, which were preferentially expressed under microoxic conditions. As expected, proteins involved in oxidative stress remediation also were induced under hyperoxic conditions. We also generated the first inventory of previously unknown small RNAs (sRNA), including untranslated regions (UTRs) and intergenic noncoding RNAs (ncRNAs), many of them differentially expressed upon O_2_ perturbation. The sRNAs that showed a strong induction were further investigated to predict their potential targets and their involvement in adaptation to these stress conditions. In this first systems-level study to include sRNAs performed on *Acaryochloris*, we uncover new insights on the particularities of “oxygenic” photosynthesis and its coevolutionary “anaerobic” metabolism.

## Materials and Methods

### Culture conditions

*A. marina* MBIC11017 was routinely kept in a culture room at 27° under 15–30 µmol photons m^−2^ s^−1^ of cool white light. Sterilized K+ES (artificial seawater), buffered with 25 mM TES at pH 8.0 was used as the culture medium for all three treatment groups. To make sure photosynthesis was not limited by CO_2_, NaHCO_3_ was dissolved in a small volume of autoclaved medium, and injected into the enclosed culture flasks every 2 d (yielding an initial concentration of 0.375 mM). The initial cell density of all culture groups was adjusted to an optical density at 750 nm of 0.2. The cultures were shaken on an orbital flat-bed shaker at ∼90 rpm.

Cultures under normal O_2_ levels were inoculated in 1-liter Erlenmeyer glass flasks containing 500 ml of medium, capped with a cotton stopper that permitted gas exchange. Thus, the O_2_ concentration of the gas phase inside the bottle was similar to atmospheric levels ∼21% (v/v).

Microoxic conditions were achieved by using a 500-ml two-necked round-bottom flask sealed tightly by a rubber stopper. Cultures were vacuumed, and refilled with pure nitrogen gas (99.95% purity) to ensure normal atmospheric pressure. We repeated this process several times, yielding a final O_2_ concentration of <0.2% inside the sealed culture flask. To maintain a microoxic condition, a positive pressure was created by bubbling nitrogen through the culture.

A similar set-up was used for hyperoxic conditions, with the exception that pure O_2_ gas was used to refill the flask after vacuuming. Because the cells generate O_2_ under illumination, ongoing input of O_2_ gas to maintain the high-oxygen concentration was not required. However, to avoid pressure build-up, the flask was revacuumed and refilled with O_2_ gas every 48 hr, as described above. The O_2_ concentration inside the flask remained within the range of 65–75% (v/v) during the experiment.

### Total RNA extraction

*Acaryochloris* cultures were harvested after 7 d from all three different treatments. The harvested cell pellets were mixed with TRIzol (TRIzol Reagent, Life Technologies, Australia), and frozen immediately using liquid nitrogen. They were stored at −80° for at least 60 min. The frozen samples were thawed in a water bath at 37°, and spun down at 16,000 × *g* for 5 min to eliminate cellular debris. This supernatant was mixed at a volume ratio of 4:1, with chloroform, and spun down at 16,000 × *g* for 10 min at 4°. The upper layer, containing RNA and DNA, was carefully transferred to a new tube, without disturbing the white middle layer of solid components. The RNA was precipitated by addition of an equal volume of isopropanol, and incubated at −20° for at least 45 min. RNA pellets were washed with 70% (v/v) ethanol, and the DNA removed using the Baseline-ZERO DNase kit (Epicentre, WI), following the manufacturer’s instructions. Prior to RNA quality assessment, the absence of DNA was confirmed by polymerase chain reaction (data not shown).

The quality of RNA was assessed on a 2100 Bioanalyzer (Agilent Technologies, CA), using a RNA 6000 Nano RNA Kit (Agilent Technologies), to obtain an RNA integrity number of >8.0. Transcripts corresponding to rRNAs (5S, 16S, and 23S) were reduced from the samples with a Ribo-Zero Kit (Epicentre), following the manufacturer’s instructions. Further processing, including quality assessment, was undertaken prior to RNAseq analysis by Beijing Genomics Institution, China.

### RNAseq data analysis

FASTQ files of the resulting sequences were processed using open source software. FASTQ files were aligned against the *A. marina* MBIC11017 reference genome on NCBI (http://www.ncbi.nlm.nih.gov/), using the “Tophat for Illumina” tool available in the Galaxy suite (Afgan *et al.* 2016). The BAM files obtained were superposed on the genome. and visualized in Artemis (Rutherford *et al.* 2000) to facilitate annotation of the predicted transcriptional units (TUs). The Java-based Rockhopper system (McClure *et al.* 2013) was used to process mapped sequence reads for differential analysis. The Rockhopper report, containing a summary of the number of reads aligned. is available in Supplemental Material, File S1.

### sRNAs target prediction

All sRNA sequences (including ncRNAs and UTRs) were obtained from the transcriptional coordinates generated by the Rockhopper software, after mapping the obtained reads to unannotated regions of the *Acaryochloris* genome. The target protein-coding genes were predicted using the IntaRNA algorithm (Busch *et al.* 2008), with a window of 275 nucleotides around the respective start codons (200 upstream and 75 downstream).

### Functional enrichment analysis

A standard functional enrichment analysis of differentially expressed genes (EADEG) was applied using hypergeometric tests, after Hernández-Prieto *et al.* (2012). Derived *p*-values were adjusted for multiple testing, while false discovery rates (FDR) were calculated using the Benjamini-Hochberg method. We used the gene annotation given in Cyanobase (Fujisawa *et al.* 2014) (http://genome.microbedb.jp/cyanobase/AM1), while gene associations with cellular functions are from the KEGG database (Kanehisa *et al.* 2016), and Gene Ontology (GO) terms in Uniprot (UniProt Consortium 2015) (Table S1). Our lists included genes associated with 116 KEGG pathways, and with 1196 GO terms. Only lists having a minimum of five genes annotated from the *Acaryochloris* genome (89 of 116 KEGG pathways, and 320 of 1196 GO terms) were investigated. To determine the functional composition of differentially expressed genes, enrichment was separately assessed for upregulated and downregulated genes.

### Data availability

The *Acaryochloris* strain used in this study is available as an axenic culture through the NBRC culture collection (NBRC 102967). Raw gene expression data, and processed information, is available at GEO with the accession number GSE89387. File S1 contains detailed information of all supplemental files.

## Results

### Culture growth

**Figure 1:**
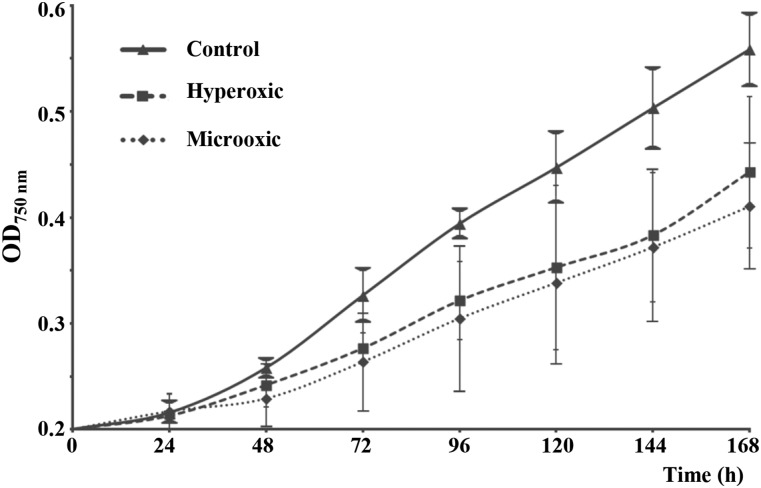
Optical density curves of *A. marina* cultures under the three different oxygen concentrations (control, microoxic, and hyperoxic). Apparent growth was monitored daily by measuring the optical density of the cultures at 750 nm (OD_750nm_). Data were averaged from quadruplicate cultures; variability in these results is represented by error bars. Apparent growth was negatively affected under both treatment conditions.

Monitoring of the O_2_ concentrations of the gas phase inside the culture bottles was performed daily with a Clark-type electrode. The concentration of O_2_ in the hyperoxic culture was maintained within the range of 65–75% (v/v), while in the microoxic culture, it was <0.2% (v/v). Thus, O_2_ concentration in the medium was equal to 350 and 1% that of air saturation at 25° for hyperoxic and microoxic cultures, respectively. These deviations from atmospheric conditions negatively affected *Acaryochloris* cells, as reflected in their low apparent growth rates (Figure 1). The control culture doubled its OD_750nm_ every ∼57 hr during the exponential growth phase, while treated cultures had doubling times of > 72 hr (Figure 1). Thus, both treatment conditions had detrimental effects on apparent cell growth, given their ∼80% decrease in growth rate (Figure 1).

### Full transcriptome profiling of Acaryochloris

RNA was extracted from cells collected after 7 d under their respective treatments, at an OD_750nm_ between 0.4 and 0.6 for all cultures. The gene expression profiling at genomic level was achieved by paired-end high-throughput sequencing of RNA, isolated from *Acaryochloris* exposed to different O_2_ concentrations. The experiment was duplicated for both test conditions, and triplicated for control conditions. To assess differential gene expression, we used the algorithms available in Rockhopper, because this software has been optimized for the analysis of RNAseq data obtained from prokaryotes (McClure *et al.* 2013). Transcript units (TUs) for 8446 of the 8462 TUs annotated for *Acaryochloris* (NCBI BioProject: PRJNA12997) were detected in both control and treated samples. Of the 16 undetected transcripts (Table S2), only one (*AM1_A0163*) has an annotated function in Cyanobase (as at June 2016), five were localized in the main chromosome, and the rest in the plasmids (two in pREB1, two in pREB2, three in pREB3, two in pREB4, and two in pREB5). RNA extracted from *Acaryochloris* cells grown under control culture conditions was used as a reference to evaluate transcript changes related to altered O_2_ levels. Under control conditions, the 50 most abundant transcripts correspond to 21 sRNAs (16 newly described in this study), and 29 to protein-coding genes, of which 12 encode subunits of PSI or PSII. Eight of these genes encoding photosystem subunits were among the 50 top transcripts in all three samples: five of them (*psaA*, *psaB*, *psaC*, *psaJ*, and *psaM*) encoding PSI subunits, two (*psbA* and *psbK*) encoding PSII subunits, and, intriguingly, a high-light induced protein (HLIP) involved in the incorporation of chlorophyll in newly assembled photosystems (Hernandez-Prieto *et al.* 2011) (Table 1).

**Table 1:** List of the top 50 most expressed open reading frames in *Acaryochloris marina* under control conditions

Gene	Product	Coordinates	Expression Control	Expression Microoxic	Expression Hyperoxic
AM1_6414*^a^*	10Sa RNA (tmRNA), ssrA	1113343, 1113638	8,862,827	1,108,900	6,593,223
AM1_NC230*^a^*	Intergenic sRNA	3815042, 3815332	1,522,785	95,333	2,440,135
AM1_6418*^a^*	RNA subunit of RNase P, rnpB	1701315, 1701673	235,615	105,408	245,204
AM1_NC208*^a^*	Intergenic sRNA	3587742, 3587961	118,457	96,521	21,716
AM1_0390	Hypothetical protein	361900, 361736	96,439	7619	297,265
AM1_4558	Hypothetical protein	4591968, 4591819	89,452	20,593	92,833
AM1_NC288*^a^*	3′UTR of AM1_4558	4591770, 4591805	65,792	8885	58,129
AM1_NC36*^a^*	5′UTR of AM1_0390	361932, 361942	64,575	6428	372,487
AM1_5793	Hypothetical protein	5875753, 5875893	63,417	22,352	169,796
AM1_1154	DNA-binding protein HU	1122737, 1123012	51,219	23,994	42,409
AM1_1660	PSI subunit, PsaC	1638289, 1638044	47,437	16,284	29,939
AM1_6426	PSI subunit, PsaM	3783989, 3783894	45,473	11,569	21,277
AM1_6419*^a^*	6Sa RNA, ssaA	3661451, 3661282	44,034	17,418	19,246
AM1_1530	Hypothetical protein	1513788, 1513489	43,502	25,195	53,880
AM1_1942	Hypothetical protein	1936231, 1936359	40,184	17,863	30,491
AM1_NC106*^a^*	5′UTR of AM1_1530	1513809, 1513828	37,734	8185	61,960
AM1_1140	Hypothetical protein	1111163, 1111005	35,060	16,626	27,461
AM1_NC37*^a^*	Intergenic sRNA	364322, 365501	29,258	8326	48,497
AM1_3851	PSII subunit, PsbK	3905041, 3905178	29,102	10,430	19,366
AM1_NC319*^a^*	Intergenic sRNA	5411225, 5411416	25,090	4405	28,342
AM1_1011	PSII protein, PsbZ	979177, 978989	24,093	14,982	14,471
AM1_NC86*^a^*	5′UTR of AM1_1114	1092698, 1092793	23,484	13,326	10,160
AM1_3193	High light inducible protein	3230482, 3230634	23,357	10,335	195,987
AM1_0039	Hypothetical protein	41120, 40992	23,217	9864	14,083
AM1_1507	Hypothetical protein	1495267, 1495127	22,795	8451	18,798
AM1_NC294*^a^*	Intergenic sRNA	4826591, 4826827	22,636	27,816	31,549
AM1_2457	PSI core protein, PsaA	2472897, 2475158	21,958	8776	27,807
AM1_NC24*^a^*	3′UTR of AM1_0345	317866, 318113	21,566	4680	23,373
AM1_2458	PSI core protein, PsaB	2475181, 2477391	20,685	5754	15,938
AM1_NC233*^a^*	5′UTR of AM1_3627	3686575, 3686602	19,985	2682	36,489
AM1_NC70*^a^*	Intergenic sRNA	1198215, 1198479	19,545	4002	2138
AM1_3627	Hypothetical protein	3686477, 3686334	19,092	7894	16,758
AM1_2630	Cyt b559 alpha subunit, PsbE	2668907, 2668656	18,427	7885	11,051
AM1_NC245*^a^*	3′UTR of AM1_3885	3937055, 3937138	18,324	4911	8432
AM1_NC154*^a^*	5′UTR of AM1_2252	2259432, 2259766	17,851	34,718	5494
AM1_2889	PSII D1 protein, PsbA	2929355, 2928273	16,679	7503	41,497
AM1_NC120*^a^*	5′UTR of AM1_1660	1638298, 1638415	16,076	7311	6156
AM1_1114	Conserved hypothetical protein	1092691, 1092497	15,015	5172	13,010
AM1_NC126*^a^*	Intergenic sRNA	1742101, 1742419	14,826	27,364	447
AM1_3119	Conserved hypothetical protein	3148026, 3147655	14,618	8294	15,236
AM1_1439	PSI protein, PsaJ	1430979, 1430824	13,098	7467	13,772
AM1_5515	Ferredoxin, 2Fe-2S type, PetF1	5563740, 5564039	12,482	11,721	9641
AM1_3950	Hypothetical protein	4000275, 4000505	12,345	10,183	16,966
AM1_1440	PSI protein, PsaF	1431487, 1430984	12,016	5467	9543
AM1_5512	PSII protein, PsbH	5561953, 5562168	10,385	3393	8835
AM1_4405	Hypothetical protein	4432868, 4432984	10,120	1755	8134
AM1_3885	Cytochrome c550, PsbV	3936566, 3937054	9500	6062	11,959
AM1_1813	Conserved hypothetical protein	1801209, 1801415	9417	9037	5358
AM1_6421*^a^*	23S ribosomal RNA	5638205, 5641084	6111	2859	20,215
AM1_6416*^a^*	23S ribosomal RNA	1408620, 1405741	6110	2859	20,215

Coordinates of the transcripts are given to facilitate the location of the noncoding sRNAs. Expression values refer to RPKM normalized by the upper quartile of gene expression. UTR, untranslated region; PSI, Photosystem I; PSII, Photosystem II.

a Rows corresponding to sRNAs.

### Identification and classification of differentially expressed transcripts

A total of 8446 transcripts (99.8% of the genes annotated in the Cyanobase database) were detected as expressed in at least one of the test conditions. Imposing a stringent threshold for minimum expression in 50 reads, we identified 6635 protein-coding and 523 noncoding TUs as expressed, under at least one of the test conditions. We considered a transcript to be differentially expressed when it had an absolute log_2_FC (fold change) value ≥1.0 (*i.e.*, a minimum twofold up or downregulation change). It is important to remark here that, since RNA sequencing data do not provide information on whether the differences in expression reflect induction or repression of transcription or changes in RNA stability under the new conditions, we use the term expression to indicate the number of detected transcripts. Using this FC criterion, 2896 TUs were identified as differentially expressed for at least one of the test conditions. These TUs consisted of 2536 mRNAs, 41 tRNAs, six rRNAs, three RNAs involved in RNA processing, and 310 unannotated sRNA, of which 248 were encoded in the chromosome (Table S3). Since the samples were treated to remove rRNAs (*Materials and Methods*), we eliminated data corresponding to 5S, 16S, and 23S rRNAs from our analysis, on the basis that differences in rRNA may reflect processing.

**Figure 2:**
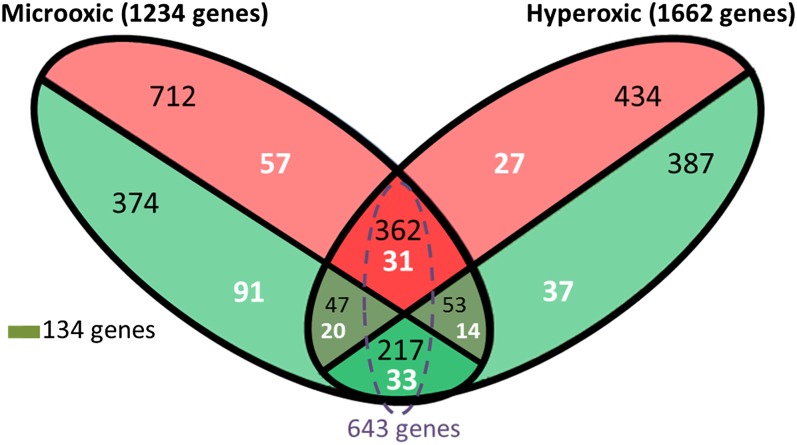
Venn diagram showing transcriptional units differentially expressed under microoxic and hyperoxic conditions. The red and green ellipsoid areas represent genes up regulated and downregulated, respectively. The number of differentially expressed sRNAs is shown in white, while the number of mRNAs is shown in black. The sum of some areas is shown to facilitate understanding of our results.

Of these 2896 TUs (1234 in microoxic, and 1662 in hyperoxic), 2119 had a significant expression change in only one of the test conditions, 643 showed a similar response under both, while 134 had opposing expression under microoxic *vs.* hyperoxic environments (Figure 2). Interestingly, among the genes with opposed expression profiles, five genes (two of them colocalized in the same genomic region) encode proteins involved in the metabolism of tetrapyrrole molecules (Table 2). Two out of five accumulated mainly under microoxic conditions (Log_2_FC >5), including *AM1_0465* encoding the oxygen-dependent Mg-protoporphyrin IX monomethyl ester cyclase (AcsF), and *AM1_0466* encoding a heme oxygenase (HO). In contrast, the transcripts of the genes encoding the three subunits of the light-independent protochlorophyllide reductase (ChlN, *AM1_1444*, ChlL, *AM1_1445*, and ChlB, *AM1_1539*) were significantly reduced under microoxic conditions. Similarly, the gene *AM1_4366*, encoding the uroporphyrin-III C-methyltransferase (cysG) at the branching point for the B12 (cobalamin) synthetic pathway, was significantly reduced under microoxic conditions. Another group of genes (*AM1_1222*, *AM1_1223*, and *AM1_1224*) in which expression was reduced under microoxic conditions, was the operon (SufBCD) coding for the proteins involved in the assembly of iron-sulfur clusters (Shen *et al.* 2007) (Table 2).

**Table 2:** Expression levels of genes discussed in the text

Gene	Product	Expression Control	Expression Microoxic	Expression Hyperoxic	Log_2_ (Microoxic/Control)	Log_2_ (Hyperoxic/Control)
AM1_4394	PSI assembly protein, Ycf37	534	433	525	−0.30	−0.02
AM1_2827	PSI assembly protein, Ycf3	971	636	649	−0.61	−0.58
AM1_1082	PSI assembly protein, Ycf4	319	346	297	0.12	−0.10
AM1_2457	PSI core protein, PsaA	27,521	11,603	45,090	−1.25	0.71
AM1_2458	PSI core protein, PsaB	25,295	8553	27,164	−1.56	0.10
AM1_1660	PSI ferredoxin protein, PsaC	79,831	18,946	44,342	−2.07	−0.85
AM1_5144	PSI protein, PsaD	12,271	3740	6069	−1.71	−1.02
AM1_2503	PSI protein, PsaE	13,114	3720	4762	−1.82	−1.46
AM1_1440	PSI protein, PsaF	18,274	6802	16,128	−1.43	−0.18
AM1_1439	PSI protein, PsaJ	26,686	10,852	24,983	−1.30	−0.10
AM1_1120	PSI protein, PsaK	5411	2674	2936	−1.02	−0.88
AM1_1637	PSI protein, PsaK	5345	2635	3063	−1.02	−0.80
AM1_1437	PSI protein, PsaL	13,970	3888	6404	−1.84	−1.13
AM1_6426	PSI protein, PsaM	85,093	14,553	27,494	−2.55	−1.63
AM1_0448	PSII D1 protein, PsbA	40	251	25	2.62	−0.66
AM1_2166	PSII D1 protein, PsbA	14,425	6869	42,113	−1.07	1.55
AM1_2889	PSII D1 protein, PsbA	19,376	5675	65,901	−1.77	1.77
AM1_2026	PSII CP47 protein, PsbB	10,087	2155	6568	−2.23	−0.62
AM1_1084	PSII CP43 protein, PsbC	4186	1012	4094	−2.05	−0.03
AM1_4084	PSII D2 protein, PsbD	9385	4764	26,427	−0.98	1.49
AM1_1083	PSII D2 protein, PsbD	9452	4747	19,155	−0.99	1.02
AM1_6045	PSII D2 protein, PsbD	42	12	45	−1.73	0.10
AM1_1130	Cytochrome b559 alpha subunit, PsbE	47	21	33	−1.13	−0.50
AM1_2630	Cytochrome b559 alpha subunit, PsbE	34,459	11,070	17,313	−1.64	−0.99
AM1_1129	Cytochrome b559 beta subunit, PsbF	20	20	20	0.00	0.00
AM1_5512	PSII 10 kDa phosphoprotein, PsbH	14,928	3783	12,054	−1.98	−0.31
AM1_3799	PSII protein, PsbI	9360	3927	4252	−1.25	−1.14
AM1_2629	PSII protein, PsbJ	7734	5426	17,622	−0.51	1.19
AM1_3851	PSII protein, PsbK	55,059	12,799	28,992	−2.10	−0.93
AM1_6425	PSII subunit, PsbL	9796	4483	11,606	−1.13	0.24
AM1_2024	PSII protein, PsbM	1021	128	465	−2.99	−1.13
AM1_5511	PSII protein, PsbN	125	25	116	−2.28	−0.11
AM1_0526	PSII manganese-stabilizing protein, PsbO	6217	1533	5680	−2.02	−0.13
AM1_0613	PSII protein, PsbP	767	460	908	−0.74	0.24
AM1_3795	PSII protein, PsbQ	8045	3177	4092	−1.34	−0.98
AM1_5050	PSII protein, PsbT	247	61	97	−2.00	−1.34
AM1_G0114	PSII 12 kDa extrinsic protein, PsbU	132	312	28	1.23	−2.20
AM1_D0138	PSII 12 kDa extrinsic protein, PsbU	481	438	1245	−0.13	1.37
AM1_3966	PSII 12 kDa extrinsic protein, PsbU	5964	2305	4019	−1.37	−0.57
AM1_5046	PSII 12 kDa extrinsic protein, PsbU	687	250	52	−1.45	−3.70
AM1_3885	Cytochrome c550 subunit of PSII, PsbV	14,802	7281	18,762	−1.02	0.34
AM1_3886	Cytochrome c550 PsbV-like protein	2641	272	1332	−3.27	−0.99
AM1_2120	PSII protein, PsbX	1297	1064	644	−0.29	−1.01
AM1_2631	PSII stability/assembly factor, Ycf48	454	259	209	−0.81	−1.12
AM1_1011	PSII protein, PsbZ	18,302	14,120	6270	−0.37	−1.55
AM1_4426	PSII protein, Psb27	459	331	120	−0.47	−1.93
AM1_5552	PSII protein, Psb28	178	284	254	0.67	0.51
AM1_4891	PSII biogenesis protein, Psb29	157	169	180	0.11	0.20
AM1_C0117	R-phycocyanin-2 subunit alpha	392	67	626	−2.53	0.67
AM1_1558	Allophycocyanin alpha subunit, ApcA	17	43	83	1.29	2.22
AM1_4469	Allophycocyanin alpha subunit, ApcA	30	31	11	0.05	−1.37
AM1_5810	Allophycocyanin alpha subunit, ApcA	3	3	14	0.00	1.91
AM1_2376	Allophycocyanin beta subunit, ApcB	4936	5426	6393	0.14	0.37
AM1_C0213	Phycocyanin alpha subunit, CpcA	17,975	4996	8646	−1.85	−1.06
AM1_C0096	Phycocyanin alpha subunit, CpcA	18,218	4972	8449	−1.87	−1.11
AM1_C0099	Phycocyanin alpha subunit, CpcA	16,251	3657	3128	−2.15	−2.38
AM1_C0191	Phycocyanin alpha subunit, CpcA	16,262	3657	3158	−2.15	−2.36
AM1_C0100	Phycocyanin beta subunit, CpcB	9490	3987	5119	−1.25	−0.89
AM1_C0192	Phycocyanin beta subunit, CpcB	18,438	6232	11,198	−1.56	−0.72
AM1_C0212	Phycocyanin beta subunit, CpcB	32,682	7338	60,428	−2.15	0.89
AM1_C0098	Phycocyanin beta subunit, CpcB	42,155	8403	23,434	−2.33	−0.85
AM1_C0215	PBS 32.1 kDa linker polypeptide, CpcC	8683	2494	5084	−1.80	−0.77
AM1_C0094	PBS 32.1 kDa linker polypeptide, CpcC	8631	2477	4950	−1.80	−0.80
AM1_C0093	PBS linker protein, CpcD	19,292	6719	16,086	−1.52	−0.26
AM1_C0216	PBS linker protein, CpcD	18,769	6471	16,609	−1.54	−0.18
AM1_C0118	Phycocyanobilin lyase subunit alpha, CpcE	438	255	500	−0.78	0.19
AM1_C0272	Phycocyanobilin lyase subunit beta, CpcF	1156	731	971	−0.66	−0.25
AM1_C0203	PBS rod-core linker polypeptide, CpcG	2103	933	1711	−1.17	−0.30
AM1_C0092	PBS rod-core linker polypeptide, CpcG	4398	1669	1772	−1.40	−1.31
AM1_C0102	PBS rod-core linker polypeptide, CpcG	4182	1090	2122	−1.94	−0.98
AM1_0450	Rieske iron-sulfur (cyt b6f) fusion protein	16	276	10	4.03	−0.63
AM1_1552	Transcriptional regulator, ChlR	21	82	58	1.92	1.42
AM1_0465	Oxygen-dependent MPE-cyclase, AcsF	16	1406	6	6.37	−1.28
AM1_0466	Heme oxygenase	33	1266	7	5.22	−2.09
AM1_1444	D-POR, ChlN	720	126	940	−2.51	0.38
AM1_1445	D-POR, ChlL	1657	421	3533	−1.97	1.09
AM1_1539	D-POR, ChlB	1659	417	2684	−1.99	0.69
AM1_4366	Uroporphyrin-III C-methyltransferase, CysG	195	92	430	−1.08	1.14
AM1_2801	Protein with homology to HemJ	182	178	162	−0.03	−0.17
AM1_0467	O_2_-independent coproporphyrinogen III oxidase, HemN	6	822	5	6.88	−0.22
AM1_1283	O_2_-independent coproporphyrinogen III oxidase, HemN	53	46	44	−0.20	−0.26
AM1_0615	Coproporphyrinogen III oxidase, aerobic, HemF	192	103	102	−0.89	−0.91
AM1_2295	Oxygen-dependent MPE-cyclase, AcsF	3331	1722	3430	−0.95	0.04
AM1_1959	Ferrochelatase, HemH	74	57	50	−0.37	−0.56
AM1_C0204	Ferrochelatase, HemH	946	788	762	−0.26	−0.31
AM1_C0107	Ferrochelatase, HemH	907	729	745	−0.31	−0.28
AM1_3193	High light inducible protein, HLIP	21,383	4241	38,036	−2.33	0.83
AM1_3366	High light inducible protein, HLIP	2	16	2	2.50	0.00
AM1_1222	FeS assembly protein, SufD	123	17	273	−2.78	1.14
AM1_1223	FeS assembly ATPase, SufC	416	80	2067	−2.36	2.31
AM1_1224	FeS assembly protein, SufB	177	30	745	−2.52	2.07
AM1_5239	Copper/Zinc superoxide dismutase, SodCC	38	100	27	1.37	−0.48
AM1_2962	Mn/Fe-containing superoxide dismutase, Sod	98	168	126	0.77	0.36
AM1_3669	Mn/Fe-containing superoxide dismutase, Sod	1061	1004	1779	−0.08	0.75
AM1_0511	Ni-containing superoxide dismutase, SodN	2744	1868	4456	−0.55	0.70
AM1_3715	Catalase/peroxidase HPI, KatG	122	55	1118	−1.14	3.19
AM1_3681	Glutathione-disulfide reductase, Gor	88	62	168	−0.50	0.93
AM1_A0300	Peroxidase/ antioxidant protein	40	21	44	−0.90	0.13
AM1_0449	Rhodanese domain protein	31	281	0	3.14	−5.00
AM1_0451	Conserved hypothetical protein	3	276	0	6.11	−2.00

Expression values refer to RPKM normalized by the upper quartile of gene expression. MPE, Mg-protoporphyrin IX monomethyl ester; D-POR, protochlorophyllide reductase; PSI, Photosystem I; PSII, Photosystem II; PBS, Phycobilisome.

### Functional composition of the set of differentially expressed genes

**Figure 3:**
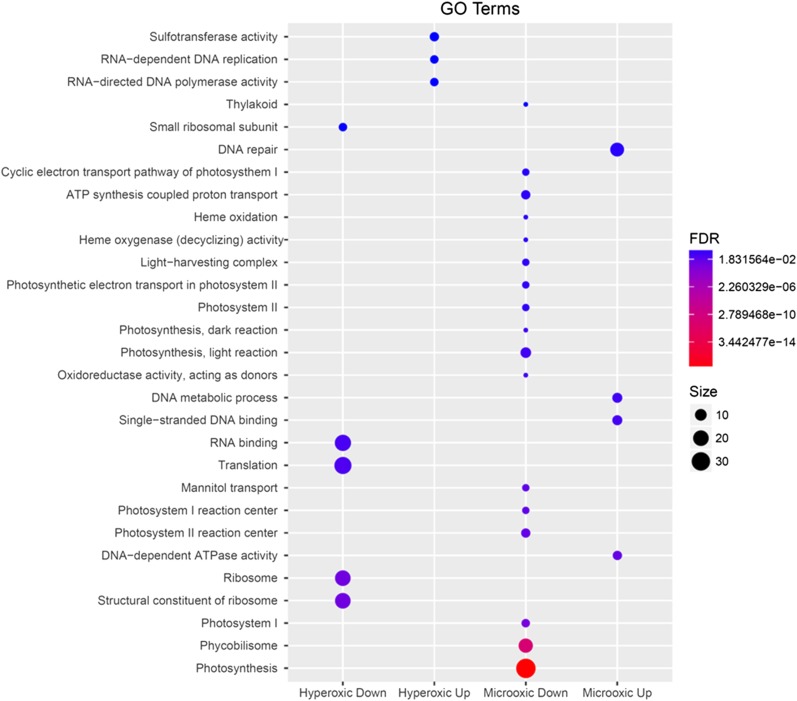
Representation of the results obtained after a standard functional enrichment analysis of differentially expressed genes using GO terms. Only categories with a FDR < 0.1 are shown (all other results are available in Table S4). The size of the circles is proportional to the number of genes in that category, reflecting differential expression, while color indicates their confidence level or FDR value. The graph was generated using the R package ggplot2.

An EADEG was applied to identify functional categories significantly affected by our treatments. After a preliminary examination of the categorical classifications available in the Cyanobase, KEGG, and GO databases, we elected to use the KEGG and GO databases for categorization, because they cover a larger number of genes compared to Cyanobase (Table S1). The results for both databases indicated that, under microoxic conditions, a significant number of genes encoding subunits of both photosystems, as well as phycobilisomes (PBS) are downregulated, having a FDR <0.05 (Table S4). The results using the KEGG database categories did not show any significant upregulation for categories, while using the GO database classification, DNA processing reactions showed a significant upregulation under microoxic conditions (Figure 3). Although increased concentrations of O_2_ in the medium caused a significant downregulation of genes encoding subunits of the ribosome, and proteins involved in RNA translation, none of these categories were significantly upregulated (Figure 3). Nevertheless, these results should be viewed with caution, since >57% of the proteins-coding genes in *Acaryochloris* lack any annotated function, *i.e.*, they are not categorized under any biological function.

### Protein-coding genes differentially expressed within significantly affected categories

Based on results of our EADEG, genes encoding proteins involved in light harvesting, and subunits of both photosystems, were among the most affected by altered O_2_ levels in the cultures. None of the genes encoding PSI subunits showed a positive regulation in either microoxic or hyperoxic environments. In fact, only three genes (*ycf3*, *ycf4*, and *ycf37*) involved in PSI assembly/stability (Dühring *et al.* 2006a; Ozawa *et al.* 2009; Boudreau *et al.* 1997) showed stable expression under microoxic conditions (Table 2). A very similar downregulation was observed for genes localized in the plasmid pREB3, encoding the PBS subunits that form the phycocyanin units, and the linker proteins. Only one (*AM1_1558*, *apcA*) of the genes encoding the allophycocyanin subunits showed increased expression levels, while expression of others (*AM1_4469*, *apcA*, *AM1_5810*, *apcA*, and *AM1_2376*, *apcB*) did not change. It is important to note that expression of *apcA* under all test conditions is 100–1000 times lower than that of *apcB*, and >10,000 times lower than some of the phycocyanin-binding apo-proteins (Table 2).

**Figure 4:**
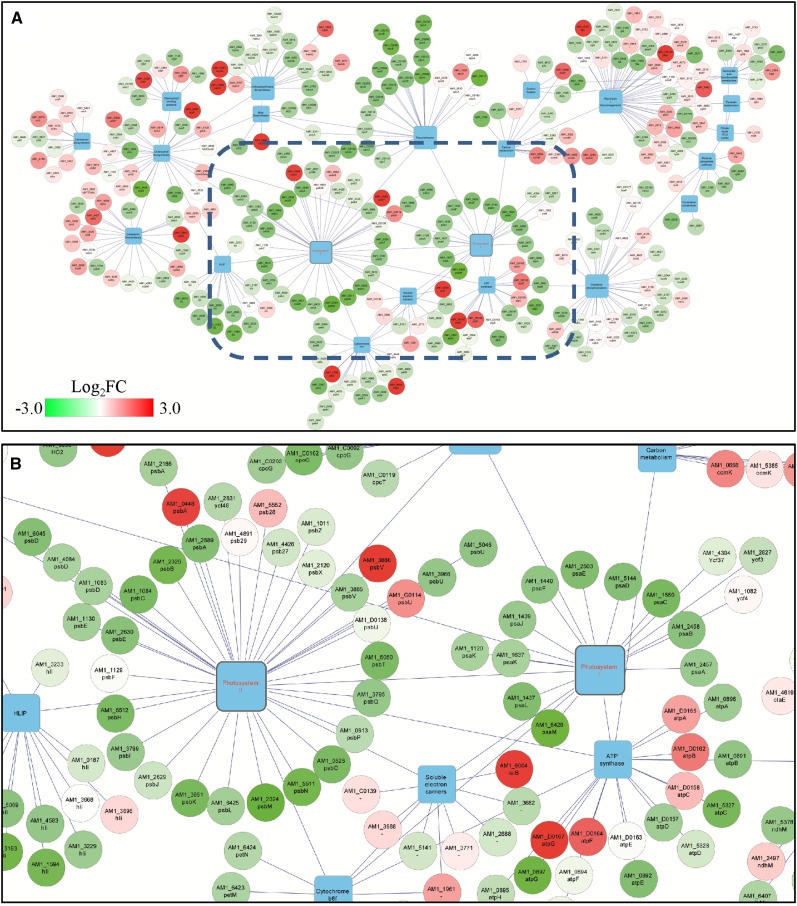
Expression data mapped onto gene network generated using Cytoscape (Lopes *et al.* 2010). Protein-coding genes (circular nodes) were linked to their associated KEGG pathway (square nodes), and colored based on their Log_2_FC (fold change) under microoxic compared with control conditions, according to the gradational color bar shown in the top panel. (A) KEGG pathways relevant to our results. The blue rectangle in (A) marks the part of the network that is enlarged in (B), showing PSI, PSII, soluble electron carriers, and ATP synthase complexes.

The expression patterns of genes coding PSII subunits were similar to those of PSI and phycobiliprotein complexes under microoxic conditions (Figure 4). Exceptions were noted for the induction of the normally cryptic *psbA1* (*AM1_0448*) gene encoding the D1 protein (Summerfield *et al.* 2008), and the expression of one of the genes (*AM1_G0114*) encoding the PsbU subunit of the oxygen-evolving complex (Figure 4). Under hyperoxic conditions, the expression of *psbA2* and *psbA3* (encoding the D1 protein) increased, as did that of the gene encoding the PsbJ subunit, and *AM1_D0138*, encoding another homolog of the PsbU subunit (Table 2).

### Expression of ROS-scavenging genes

**Figure 5:**
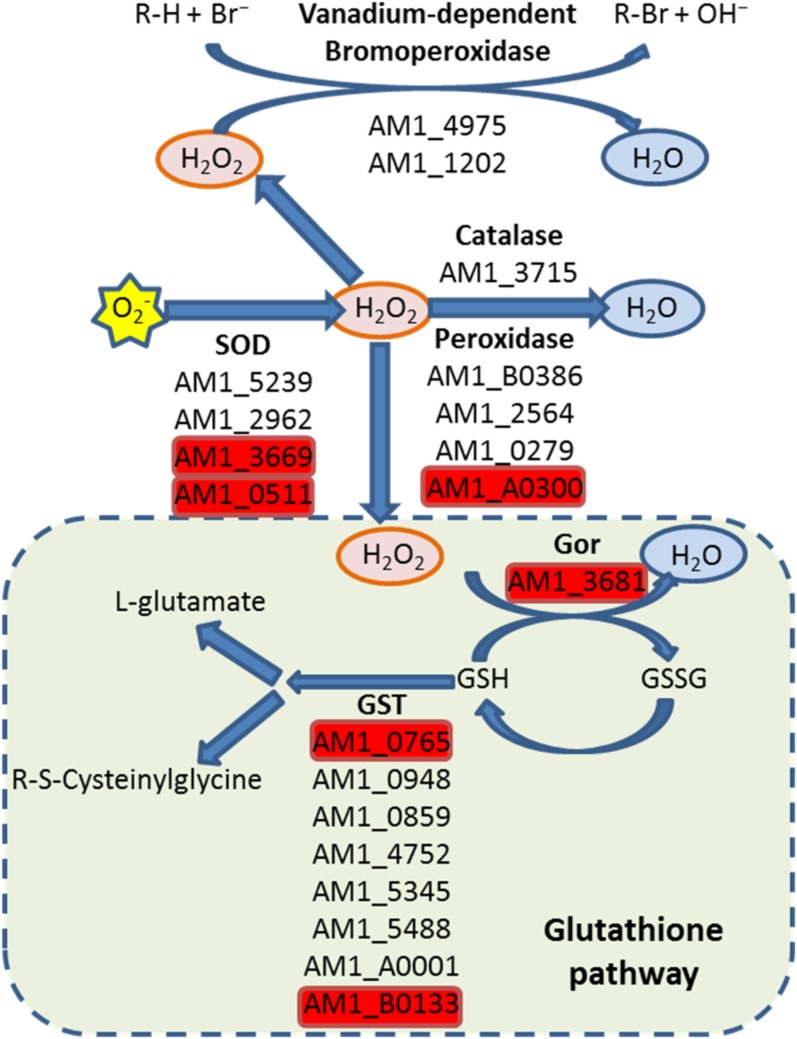
Putative ROS-scavenging pathways. Genes encoding antioxidant enzymes or involved in ROS-scavenging are shown under the reaction that they catalyze. Genes highlighted in red were identified as upregulated in the hyperoxic environment. SOD, Superoxide dismutase; Gor, Glutathione-disulfide reductase; GST, Glutathione S-transferase (GST); GSH, Glutathione; GSSG, Glutathione disulfide.

ROS are by-products of both respiration and photosynthesis. Thus, efficient scavenging is crucial to prevent photo-oxidative damage, especially in cyanobacteria, in which both processes occur simultaneously. Enzymes, like superoxide dismutase (SOD), efficiently scavenge the superoxide (O_2_^−^). In *Acaryochloris*, four open reading frames, *AM1_5239* (Cu^2+^/Zn^2+^-SOD), *AM1_2962* (Mn^2+^/Fe^2+^-SOD), *AM1_3669* (Mn^2+^/Fe^2+^-SOD), and *AM1_0511* (Ni-SOD), encode proteins resembling SOD. Of these, only *AM1_3669* and *AM1_0511* accumulated under hyperoxic conditions, but decreased under microoxic conditions (Figure 5). The highest induction of genes encoding scavenging proteins under hyperoxic conditions was for *AM1_3715* (catalase, *katG*) (log_2_FC > 3), which participates in the elimination of H_2_O_2_. Other genes, related to H_2_O_2_ scavenging, such as *AM1_A0300* (thiol-specific peroxidase), increased their expression under hyperoxic conditions, but had decreased expression under microoxic conditions. Interestingly, the genes encoding enzymes involved in glutathione metabolism either did not change markedly, or else their transcripts increased more under microoxic conditions than under hyperoxic conditions. The only gene for which expression under hyperoxic conditions was significantly higher than under control conditions was *AM1_3681*, encoding glutathione-disulfide reductase; its expression decreased with respect to the control under microoxic conditions (Figure 5).

Terminal oxidases also play an important role in ROS prevention. The presence of terminal oxidases in the thylakoid membrane is key to balancing metabolic flow between the respiratory and photosynthetic electron transport chains, as well as reducing the amount of O_2_ in the vicinity of the thylakoid membrane thus, preventing ROS (Schmetterer 2016). In *Acaryochloris*, genes encoding four terminal oxidases have been previously annotated: (i) *AM1_4621* (*coxB*), AM1_4620 (*coxA*), and AM1_4619 (*coxC*), encoding subunits of a mitochondrial-type cytochrome *c* oxidase (cox) complex; (ii) *AM1_A0138*; (iii) *AM1_0843*; and (iv) *AM1_1551*, encoding plastidic-type terminal oxidases (ptox) (Schmetterer 2016). In *Anabaena variabilis*, *coxB*, the first gene within the cox locus (*coxBAC*), was apparently transcribed more often than the other two genes in the operon (Schmetterer *et al.* 2001). A similar expression pattern is apparent in our results, with *coxB* (*AM1_4621*) transcript levels at least three times higher than the other two subunits (Figure S1A). In fact, *coxB* expression level increased up to eight times under hyperoxic conditions. The expression of *coxA* and *coxC* showed no significant changes under altered O_2_ conditions. Intriguingly, the expression of two (*AM1_A0138* and *AM1_0483*) of the three ptox genes was very low under all conditions, while the expression level of *AM1_1551* was >45 times higher under microoxic than under control conditions (Figure S1B).

### Potential transacting sRNAs involved in the adaptation to aerobic variations

Alignment of the reads resulted in a large number of sRNAs corresponding to either UTRs or intergenic ncRNAs. We differentiated sRNAs within these two main groups, based on whether the distance between the detected sRNA and the closest annotated mRNA was ≤20 nucleotides (in the case of UTRs), or >20 nucleotides (in the case of ncRNAs). The transcription start site and orientation of the transcript were determined from predictions obtained using algorithms embedded in PePPER (de Jong *et al.* 2012). Using this criterion for the 248 differentially expressed chromosome-detected transcripts, we identified 190 sRNAs as UTRs and 58 as ncRNAs. The UTR expression level was mostly correlated (Spearman correlation coefficient, *r*_S_ > 0.6) with the closest gene, except for four of the sRNAs (AM1_NC24, AM1_NC96, AM1_NC169, and AM1_NC181) (Figure S2).

**Figure 6:**
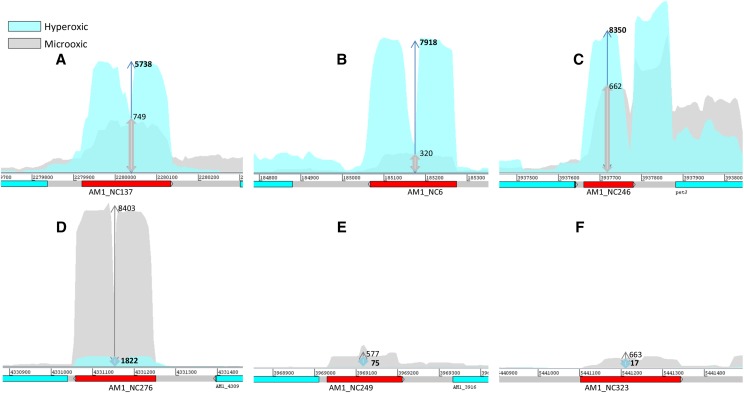
Noncoding RNAs regulated in opposing directions, under the two oxygen treatment conditions. (A–C) were induced under hyperoxic conditions; while (D–F) were induced under microoxic conditions. The arrows and the numbers adjacent to them represent relative expression. Chromosome coordinates are given above the gene representation. RPKM, reads per kilobase per million mapped reads.

Some of the intergenic ncRNAs were among the most highly expressed transcripts under all three conditions profiled (Table 1). Of the 58 differentially expressed ncRNAs localized in the chromosome, only six showed significant opposing expression profiles for microoxic and hyperoxic conditions. Three were less abundant under microoxic conditions (AM1_NC12, AM1_NC161, and AM1_NC254), while transcripts for the other three (AM1_NC270, AM1_NC256, and AM1_NC315) were enhanced in the hyperoxic culture (Figure 6). Given that most bacterial ncRNAs act through sequence-specific binding to regions close to the ribosome-binding site of mRNAs, we sought to predict potential RNA targets using a window of 275 nucleotides around their respective start codons. Setting a *p*-value threshold of 0.01 for results obtained from the IntaRNA server, the number of predicted targets was 95 for AM1_NC6, 96 for AM1_NC246, 50 for AM1_NC137, 89 for AM1_NC276, 45 for AM1_NC249, and 84 for AM1_NC323. The number of targets was much larger than expected based on available literature. To reduce these targets, as well as the number of false positives, we calculated the correlation between the numbers of reads obtained for each of the three conditions. We reasoned that any potential target should show an inverse correlation with the particular ncRNA, given that most bacterial ncRNAs act as negative regulators of gene expression, even when other mechanisms cannot be disregarded (Storz *et al.* 2011). Defining a threshold of *r*_S_ ≤ −0.5 for inverse correlation, the list of candidates was reduced to 38 potential targets for AM1_NC12, 43 for AM1_NC254, 22 for AM1_NC161, 21 for AM1_NC270, 15 for AM1_NC256, and 24 for AM1_NC315 (Table S5). Functional enrichment analyses of these targets returned significant results (FDR < 0.05) for only two ncRNAs: AM1_NC6 and AM1_NC276. Specifically, AM1_NC6 targets were significantly enriched in genes involved on “DNA integration,” while AM1_NC276 targets showed enrichment in genes encoding proteins involved in “aerobic respiration” (Table 3).

**Table 3:** Functional enrichment analysis of the predicted targets for intergenic small noncoding RNAs targets

GO ID	Function	Targets of AM1_NC276	*P*-Value	FDR
**GO:0009060**	**Aerobic respiration**	**2**	**5.29 × 10^−6^**	**0.006**
GO:0020037	Heme binding	2	0.0014	0.839
GO:0005506	Iron ion binding	2	0.0026	1
GO:0009055	Electron carrier activity	2	0.0059	1
GO:0019898	Extrinsic to membrane	1	0.0083	1
GO:0042549	Photosystem II stabilization	1	0.0083	1
GO:0009654	Oxygen evolving complex	1	0.0097	1
GO ID	Function	Targets of AM1_NC6	*P*-Value	FDR
**GO:0015074**	**DNA integration**	**5**	**5.71 × 10^−5^**	**0.048**
GO:0003676	Nucleic acid binding	5	0.0036	0.985
GO:0003952	NAD+ synthase (glutamine-hydrolyzing) activity	1	0.0074	0.985
GO:0004127	Cytidylate kinase activity	1	0.0074	0.985
GO:0004553	Hydrolase activity, hydrolyzing O-glycosyl compounds	2	0.0023	0.985
GO:0004592	Pantoate-beta-alanine ligase activity	1	0.0074	0.985

Our analysis was performed similarly to the enrichment analysis of differentially expressed genes described in the *Materials and Methods*. The top six categories with the lowest *p*-values are shown. Only GO categories having a FDR ≤ 0.05 (in bold) were considered significant.

## Discussion

In the laboratory, *Acaryochloris* can grow as a free-living form, under conditions very different from those in which it was initially isolated. In nature, it forms part of an algal mat, associated with colonial ascidians (Miyashita *et al.* 1996). It has been speculated that the multiplicity of homologous genes, and the relatively large genome size, of *Acaryochloris* reflect its evolutionary adaptation to its niche (Swingley *et al.* 2008), in contrast to the reduced genome size of the picoplanktonic *Prochlorococcus* genus (Delaye and Moya 2010; Dufresne *et al.* 2005). Here, we have shown that, under different O_2_ conditions, expression levels among homologous genes varies. Given such adaptive capability, it is tempting to speculate on the flexibility of *Acaryochloris* to adapt to changing environmental conditions. This would be one of the benefits obtained from having coexisting multiple copies of genes, in spite of the cost of such a large genome size.

**Figure 7:**
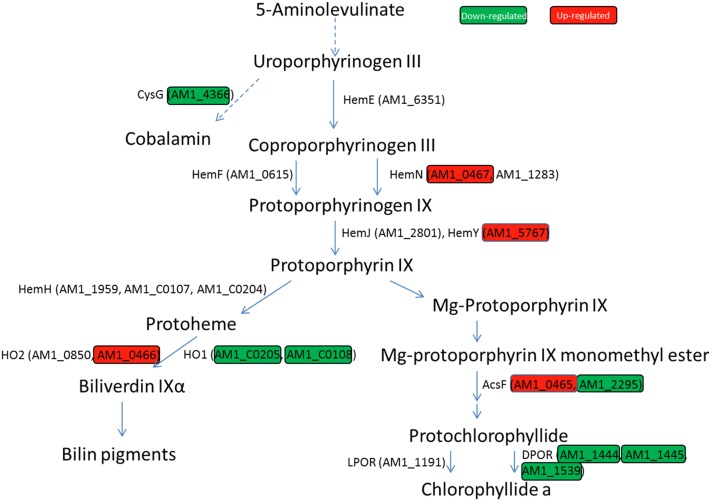
Diagram representing the tetrapyrrole biosynthetic pathway. Only genes encoding proteins discussed in the text are shown. Genes differentially expressed are highlighted in green and red, indicating downregulation and upregulation under microoxic conditions, respectively. Specific values are given in Table 2.

The combination of porphyrin-containing molecules, O_2_, and light often results in photo-oxidative damage to cellular structures. Hence, it has been speculated that divergence of the biosynthesis of bacteriochlorophyll and chlorophyll occurred to reduce photo-oxidative damage under an increasingly oxic atmosphere (Reinbothe *et al.* 1996). The syntheses of various tetrapyrrole molecules (heme, bilins, cobalamin, and chlorophyll) share a common pathway from ALA to uroporphyrinogen III. At this point, the cobalamin biosynthesis pathway branches from the uroporphyrinogen III pathway via a methylation reaction, catalyzed by the multifunctional chelatase CysG. The downregulation of *cysG* expression under microoxic conditions indicates that O_2_ levels are an important regulatory element for the synthesis of cobalamin. In fact, CysG directs the uroporphyrinogen III pathway toward the synthesis of cobalamin either via an oxygen-independent or dependent pathway (Figure 7). Alternatively, it can be redirected toward the heme or chlorophyll biosynthetic pathways. In these pathways, O_2_ levels influence the expression of genes encoding enzymes involved in oxidation. The part common to the heme and chlorophyll biosynthetic pathways, from uroporphyrinogen III to protoporphyrin IX, contains several oxidation reactions. The first oxidation step converts coproporphyrinogen III to protoporphyrinogen IX. This oxidation is catalyzed by either HemF or HemN, using O_2_ or a 5′deoxyadenosil radical generated from S-adenosylmethionine, respectively. HemN contains a [4Fe-4S] cluster, as a prosthetic group, sensitive to the presence of O_2_. In *Synechocystis*, a mutant lacking *hemF* was able to grow under microoxic conditions, but did not grow under aerobic conditions. In contrast, an *hemN* knockout mutant showed impaired growth only under microoxic conditions (Goto *et al.* 2010). Thus, in the presence of O_2_, it could be expected that *hemN* expression would be minimal compared with *hemF*. In *Acaryochloris*, the product of two genes (*AM1_0467* and *AM1_1283*) resembles HemN, but only *AM1_0467* had a strong induction (Log_2_FC ∼6.8) under microoxic conditions (Figure 7). Expression of *HemF* (*AM1_0615*) did not show any significant change. This suggests that *AM1_0467* is the homolog to *hemN* in *Acaryochloris*, based on its expression profile. The product of the HemN/HemF reaction (protoporphyrinogen IX) is converted to protoporphyrin IX, becoming the final precursor common to Chl and heme, as well as heme-derived bilins. The protoporphyrinogen IX oxidation to protoporphyrin IX can be catalyzed by three enzymes, namely HemG, HemY, and HemJ (Kato *et al.* 2010). Only HemG, which is absent in most cyanobacteria, seems to be oxygen-independent (Boynton *et al.* 2009). Most cyanobacteria use the oxygen-dependent HemJ, although a few use HemY (Kobayashi *et al.* 2014). Interestingly, *Acaryochloris* contains both a HemJ- and a HemY-like enzyme, and, like most cyanobacteria, it lacks a gene homologous to *HemG*. *AM1_5767*, having enhanced expression in the microoxic environment, encodes a protein containing a HemY-like domain. A BLAST search, using the sequence for the *Synechocystis* HemJ (encoded by *slr1790*) (Kato *et al.* 2010), returned AM1_2801 as being highly homologous to HemJ. Unlike *AM1_5767*, the expression of *AM1_2801* was not influenced by altered O_2_ levels, suggesting that the main pathway for the oxidation of protoporphyrinogen IX in *Acaryochloris* under microoxic conditions is through HemY (AM1_5767). After this step, iron or magnesium is incorporated into protoporphyrin IX by a ferro- or magnesium chelatase, leading to the synthesis of heme and bilins (in the case of iron insertion), or Chl (in the case of magnesium insertion) (Chen 2014). In the Chl branch, the next substrate that is oxidized is Mg-protoporphyrin IX monomethyl ester (MPE). This reaction is catalyzed by MPE cyclase, which converts MPE into protochlorophyllide (Beale 1999). MPE can follow two pathways: one through an oxygen-dependent MPE-cyclase (AcsF, encoded by *AM1_0465*, or *AM1_2295*); and the other through an oxygen-independent MPE-cyclase (BchE) (Raymond and Blankenship 2004). BLAST results did not return any gene homologous to BchE in *Acaryochloris*. Nevertheless, our results show that the expression of both *ascF* (*AM1_0465*, *AM1_2295*) homologs differ. *AM1_0465* was strongly induced under microoxic conditions (log_2_FC > 6), while the expression of *AM1_2295* was reduced by almost half (Figure 7). In *Synechocystis*, a similar expression profile was described for two homologous genes encoding AcsF; deletion of these genes impaired growth under aerobic conditions (Minamizaki *et al.* 2008). Based on the conclusions drawn for *Synechocystis*, it is likely that *AM1_0465* is the main MPE-cyclase in *Acaryochloris* under microoxic conditions, while, under aerobic conditions, our results show its expression is null, compared to that of *AM1_2295* (Figure 7).

Based on published work, the FeS cluster within the ChlL subunit of the light-independent protochlorophyllide reductase (D-POR) shows a high vulnerability to O_2_ (Nomata *et al.* 2006; Yamazaki *et al.* 2006). Such susceptibility might explain why this multimeric enzyme functions in the dark, when O_2_ levels are low. Intriguingly, downregulation of the genes encoding the subunits of D-POR under microoxic conditions was observed, confirming that expression levels of this gene are controlled by the reduction state of the photosynthetic electron transport chain, and not by O_2_ levels (Horiuchi *et al.* 2010). In contrast, the expression level of the light-dependent protochlorophyllide reductase (L-POR) did not change under microoxic conditions, but decreased under hyperoxic conditions (Table 2).

Because of the potential deleterious effects of a misregulation of the tetrapyrrole biosynthetic pathway, it is expected that several regulatory factors (including sRNAs) control its activity. However, how a cell senses O_2_ levels, and controls the expression of multiple genes is not fully understood. In *Synechocystis*, O_2_ levels are sensed by the transcriptional factor ChlR (*sll1512*), which positively regulates *acsF*, *ho2*, and *hemN* expression (Aoki *et al.* 2012). The homolog to ChlR in *Acaryochloris* is encoded by *AM1_1552*. Expression of *AM1_1552*, as expected for a positive regulator of genes sensitive to O_2_, increased under microoxic conditions (log_2_FC ∼1.9). A search using the FIMO tool within the MEME suite using the ChlR recognition motif (TTMCC-N_4/3_-GGWAA) provided by Aoki *et al.* (2012) returned a putative site (*p*-value <0.0005), located 22 bp upstream of *AM1_0466* (*ho2*).

Furthermore, the expression control performed by regulatory factors, the synthesis of the final products, and their assembly into the apoprotein moiety, have to be tightly regulated to avoid their accumulation as free pigments in the membrane. For example, members of the HLIP family appear to mediate between both pathways in the assembly of photosynthetic complexes (Hernandez-Prieto *et al.* 2011; Yao *et al.* 2012; Sobotka *et al.* 2008; Adamska *et al.* 2001). In *Synechocystis*, HLIPs accumulate under multiple-stress conditions, while, under laboratory growth conditions, they are expressed at a low level (He *et al.* 2001). In *Acaryochloris*, there are 13 *hlip* genes; our data showed that *AM1_3193* is among the most expressed transcripts in the control sample, contrary to what was observed in *Synechocystis* (He *et al.* 2001). In general, *hlip* expression levels decreased both under microoxic and hyperoxic conditions, following the trend observed for both photosystems. The regulatory role of HLIPs is most evident when examining the C-terminal extension of the ferrochelatase gene in cyanobacteria and chloroplasts, which shares a high homology with HLIPs (Funk and Vermaas 1999). This HLIP-like extension appears to induce or repress ferrochelatase activity, depending on the amount of free chlorophyll in the thylakoid membrane (Sobotka *et al.* 2008). In most cyanobacteria, only one gene encodes for ferrochelatase, while in *Acaryochloris* there are three ferrochelatase gene copies (*AM1_1959*, *AM1_C0107*, and *AM1_C0204*), and all of them have HLIP-like C-terminal extensions. *AM1_C0107* and *AM1_C0204*, localized in the plasmid pRBE3, encode identical ferrochelatases; and their transcripts are >100 times more abundant than those generated from the copy (*AM1_1959*) localized in the chromosome, under all test conditions. The identification of ferrochelatase, as a pivotal enzyme at the intersection between Chl and heme syntheses (Figure 7), makes the regulation of its function by the HLIP-like extension essential to the flux of metabolites toward one or other of its final products.

In cyanobacteria, a large portion of the heme generated by ferrochelatase is funneled toward heme oxygenase. This is the first step in the synthesis of bilins, the pigments bound in the phycobiliproteins of the PBS. The PBS antenna found in *Acaryochloris* consists of a single rod structure (Chen *et al.* 2009; Hu *et al.* 1999), in which phycocyanin (pc)-containing subunits are the main component, and allophycocyanin (Apc)-containing subunits are only a minor component of the bottom disc of the rod-structured phycobiliprotein complex (Marquardt *et al.* 1997). This structure explains the larger number of reads for the genes encoding the pc-binding apoproteins (Table 2), compared with the Apc-containing subunits (ApcA and ApcB). Furthermore, their gene loci are physically separated from each other, with the genes encoding the Apc proteins, ApcA (*AM1_1558*, *AM1_4469*, and *AM1_5810*) and ApcB (*AM1_2376*), localized in the main chromosome, and the ones encoding the pc-binding apoproteins localized in the pREB3 plasmid. Our results and previously published work (Lin *et al.* 2013) show that, under microoxic conditions, the expression of genes encoding PBS subunits and their assembly in the antenna complex, are significantly decreased. Similarly, experimental conditions that limit access of cultures to essential nutrients also induce a downregulation of PBS encoding genes (Foster *et al.* 2007; Wang *et al.* 2004; Zhang *et al.* 2008). Downregulation of PBS encoding genes under microoxic conditions also has been observed in *Synechocystis* sp. PCC6803 (Summerfield *et al.* 2011). This downregulation may reflect decreased formation of bilins by the oxygen-dependent heme oxygenase (HO). In most cyanobacteria, two genes encode for heme oxygenases (*ho1*, and *ho2*), with HO2 having a higher affinity for O_2_, and being most active under microoxic conditions (Aoki *et al.* 2011). In *Acaryochloris*, two genes encode proteins with high similarity to HO1 (*AM1_C0205* and *AM1_C0108*), while two others encode HO2 (*AM1_0850* and *AM1_0466*). The expression of both genes encoding for HO1 decreased under microoxic conditions, while the expression of *AM1_0466* increased under microoxic conditions (log_2_FC > 5), revealing this gene as most probably HO2.

In *Synechocystis*, it was noted that the expression of genes encoding PBS subunits is highly correlated with genes encoding subunits of the ATP synthase (ATPase) complex (Hernández-Prieto *et al.* 2016; Summerfield *et al.* 2011). Hence, a similar downregulation of ATPase subunits would also be expected for *Acaryochloris* under microoxic conditions. In *Acaryochloris*, two sets of genes encoding for ATPase subunits exist (one set is localized in the chromosome, and another in the pREB4 plasmid) (Swingley *et al.* 2008). The expression of the genes encoding ATPase located in the plasmid increased slightly under microoxic conditions, while the ATPase from the main chromosome showed decreased expression under microoxic conditions, consistent with the results for *Synechocystis* (Hernández-Prieto *et al.* 2016). In fact, the ATPase encoding genes localized in the chromosome are phylogenetically closer to ATPase from other cyanobacteria (Swingley *et al.* 2008) than those localized in the plasmid. In addition to their downregulation under microoxic conditions, the ATPase genes localized in the chromosome are transcribed more frequently under all test conditions than the copies localized in the plasmid. These results indicate that the ATPase gene copies localized in the plasmid might be cryptic, or function in conditions different from the ones tested here.

A well-documented effect observed under microoxic conditions in cyanobacteria is the induction of the *psbA1* gene encoding a homolog of the D1 protein of PSII (Summerfield *et al.* 2008), with repression of *psbA2* and *psbA3*. D1 differential expression also was confirmed in this study, consistent with previously published results (Kiss *et al.* 2012). Noticeably, a similar expression profile was observed here for one (*AM1_G0114*) of the four genes (*AM1_G0114*, *AM1_D0138*, *AM1_3966*, and *AM1_5046*) encoding the PsbU subunit of PSII (Figure 4). The PsbU subunit is associated with the oxygen-evolving complex, and functions to stabilize the PSII complex under high-intensity light conditions, protecting it from ROS (Abasova *et al.* 2011). The differential induction of this gene is interesting, since it has been assumed that, because of differences in the sequence of the *psbA1* encoded D1 protein, the PSII complexes assembled with this protein lack the capacity to evolve O_2_ (Kiss *et al.* 2012; Murray 2012). The expression of the rieske-containing subunit (PetC) (Wenk *et al.* 2005) of the cytochrome *b*_6_*f* subunits is affected in a similar manner to *psbA1*. Similar to D1, PetC can be transcribed from three genes (*AM1_4450*, *AM1_0450*, and *AM1_1961*), with *AM1_0450* being the only one induced under microoxic conditions (Table 2). It is important to note that *AM1_0450* is upstream of *psbA1*. Thus, it is likely that they are cotranscribed in *Acaryochloris*, together with another two genes encoding proteins of unknown function: one (*AM1_0449*) containing a bacteria-conserved domain (DUF2892), and the other (*AM1_0451*) containing a rhodanese-like domain linked with assimilation of thiosulfate under anaerobic conditions in other bacteria (Schedel and Trüper 1980). A similar gene cluster is observed in *Synechocystis* genome, where *psbA1* (*slr1181*) is the first gene of a set of 10 genes orientated in the same direction, including *slr1184* encoding a rhodanese-like protein, and *slr1185* encoding PetC; expression levels in these genes were also higher under microoxic conditions (Summerfield *et al.* 2008). Based on the microarray meta-analysis presented in CyanoEXpress, only the expression of *slr1182*, *slr1183*, and *slr1184* seem to follow the same trend under different environmental conditions, as expected for genes in an operon (Hernández-Prieto *et al.* 2016). Nevertheless, results obtained from genes for which expression levels are low under most conditions (as is the case for *psbA1*) should be viewed with caution, especially when interpreting correlations. Further investigation to confirm whether expression of these proteins results in a restructuring of the photosynthetic complexes, and rerouting of the electron transport chain under microoxic conditions, is needed, but is beyond the scope this paper.

Transcriptome data (Mitschke *et al.* 2011; Kopf *et al.* 2014; Voigt *et al.* 2014; Hernández-Prieto *et al.* 2012), as well as computational predictions (Voß *et al.* 2009; Voigt *et al.* 2014), of sRNAs in cyanobacteria have revealed a large number of previously unidentified noncoding protein transcripts, exceeding all previous predictions. Although the roles of some of these sRNAs have been partially characterized in cyanobacteria (Nakamura *et al.* 2007; Voß *et al.* 2007; Dühring *et al.* 2006b), the function of most of them is still unknown. A well understood process in *Escherichia coli* is the degradation of complementarily paired ncRNAs and mRNAs, mediated by the protein Hfq (Massé *et al.* 2003). Such processes affect protein synthesis at the transcriptional level, saving valuable resources that can be used to synthesize a different protein complement that is more suitable to the new environmental conditions. In cyanobacteria, a gene encoding a homolog to Hfq has been identified, but its role in ncRNA/mRNA degradation has not yet been demonstrated (Bøggild *et al.* 2009; Dienst *et al.* 2008). Of the 58 ncRNAs localized in the chromosome (Table 1), only six (AM1_NC12, AM1_NC161, AM1_NC254, AM1_NC270, AM1_NC256, and AM1_NC315) showed significant opposing expression profiles for microoxic and hyperoxic conditions, as would be expected for ncRNAs involved in adaptation to different O_2_ levels (Figure 6). Of these, functional enrichment analyses of their potential targets returned significant results (FDR < 0.05) for only two ncRNAs: AM1_NC6 and AM1_NC276. The targets predicted for AM1_NC276 comprised genes within the category “aerobic respiration” (Table 3), indicating that expression of this ncRNA might be relevant for adaptation to altered O_2_ levels. In *E. coli*, several ncRNAs have been shown to play a role in adaptation to oxidative stress (Berghoff and Klug 2012), but further experimental work is necessary to determine whether they have the same functions in *Acaryochloris*.

In conclusion, the large number of protein-coding genes and sRNAs detected as differentially expressed under our test conditions revealed that there is a high level of regulation related to O_2_ in cyanobacteria. The multiplicity of genes encoding homologous proteins in *Acaryochloris* exceeds that of many cyanobacteria, indicating a complex regulatory network in this organism. Multiple ROS-scavenging pathways, and their different transcriptomic responses, may represent the history of alternative ROS-scavenging mechanisms, which has evolved and developed in parallel to new metabolic pathways that produce ROS.

Ultimately, the lack of efficient methods to generate mutants in *Acaryochloris* makes environmental studies, such as this one, key to understanding its regulation and annotating its yet unknown gene functions.

## Supplementary Material

Supplemental material is available online at www.g3journal.org/lookup/suppl/doi:10.1534/g3.116.036855/-/DC1.
