# Supplementary material for: The Complex Transcriptional Response of Acaryochloris marina to Different Oxygen Levels
Source: G3 (Bethesda). 2016 Dec 14;7(2):517–32. doi: 10.1534/g3.116.036855 (PMC5295598; doi:10.1534/g3.116.036855)
Supplement: Supplementary file 1 [file 517FigureS1.pptx]

## Slide 1
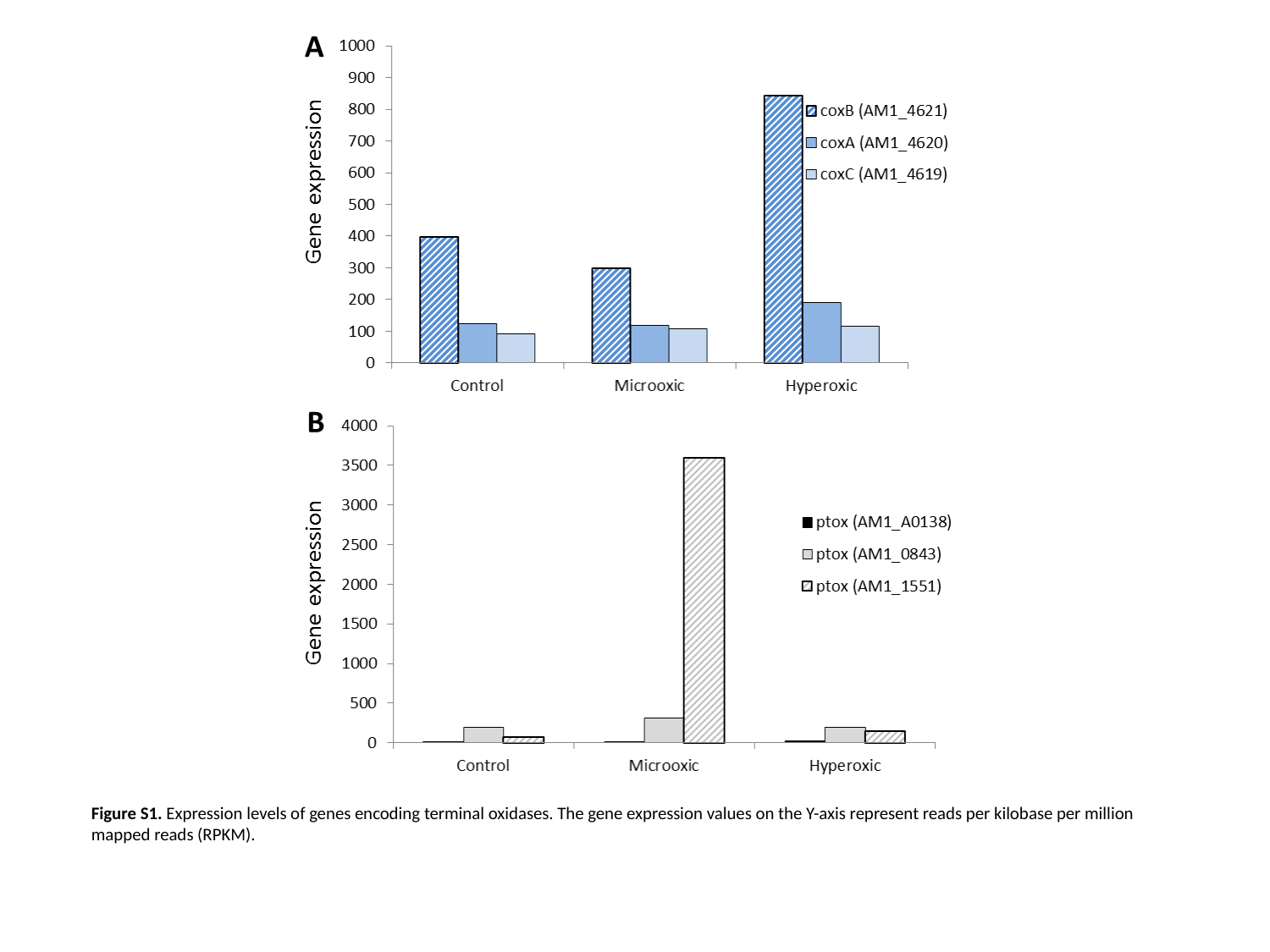

Figure S1. Expression levels of genes encoding terminal oxidases. The gene expression values on the Y-axis represent reads per kilobase per million mapped reads (RPKM).
