# Supplementary material for: The Complex Transcriptional Response of Acaryochloris marina to Different Oxygen Levels
Source: G3 (Bethesda). 2016 Dec 14;7(2):517–32. doi: 10.1534/g3.116.036855 (PMC5295598; doi:10.1534/g3.116.036855)
Supplement: Supplementary file 2 [file 517FigureS2.pptx]

## Slide 1
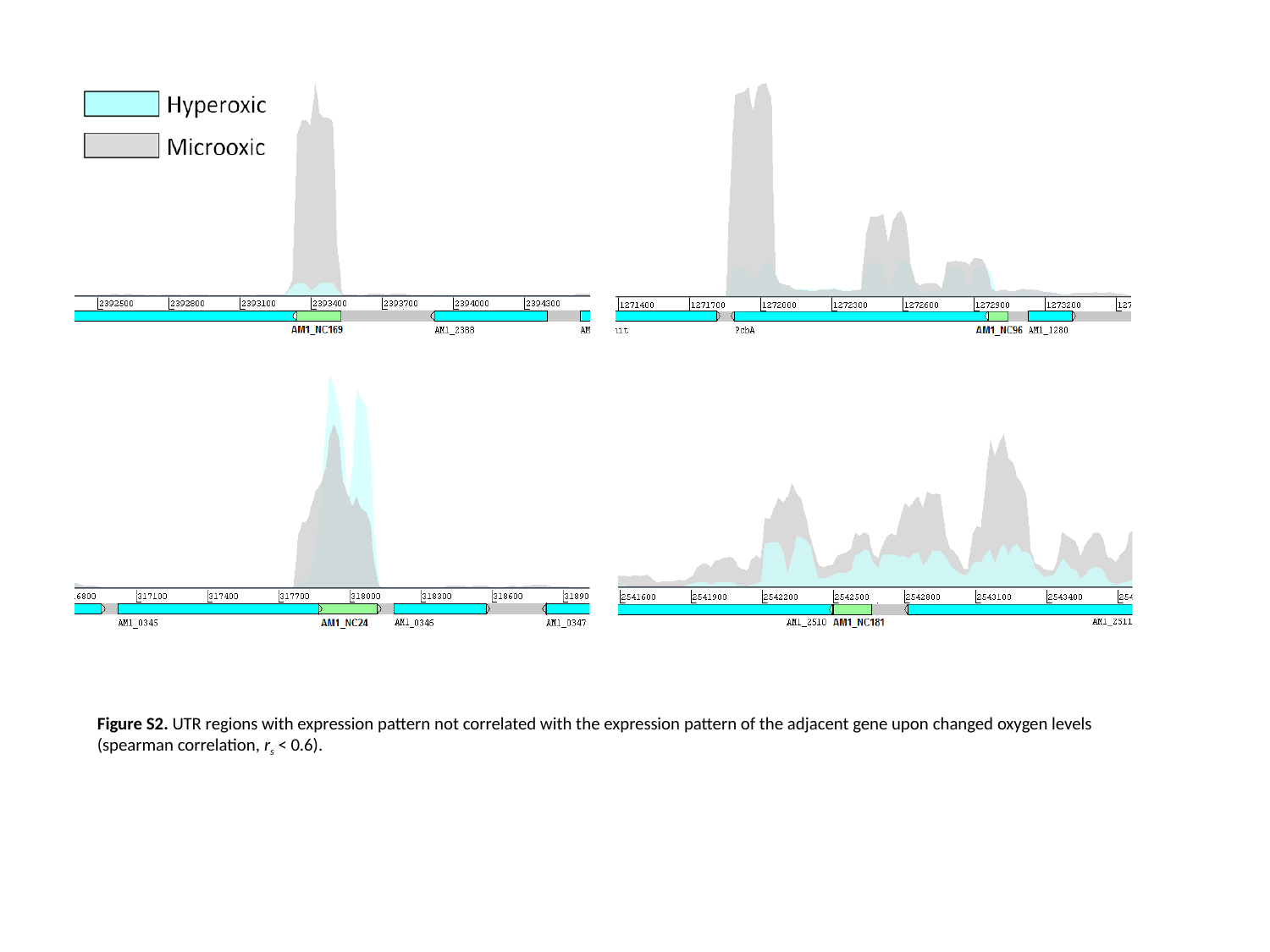

Figure S2. UTR regions with expression pattern not correlated with the expression pattern of the adjacent gene upon changed oxygen levels (spearman correlation, rs < 0.6).
