# Supplementary material for: The Complex Transcriptional Response of Acaryochloris marina to Different Oxygen Levels
Source: G3 (Bethesda). 2016 Dec 14;7(2):517–32. doi: 10.1534/g3.116.036855 (PMC5295598; doi:10.1534/g3.116.036855)
Supplement: Supplementary file 3 [file 517TableS1.docx]

Table S1. Genes lists within functional categories and subcategories represented in CyanoBase, KEGG pathways and Gene Ontology (GO). (.xlsx, 611 KB)

<http://www.g3journal.org/lookup/suppl/doi:10.1534/g3.116.036855/-/DC1/TableS1.xlsx>
