# Supplementary material for: The Complex Transcriptional Response of Acaryochloris marina to Different Oxygen Levels
Source: G3 (Bethesda). 2016 Dec 14;7(2):517–32. doi: 10.1534/g3.116.036855 (PMC5295598; doi:10.1534/g3.116.036855)
Supplement: Supplementary file 5 [file 517TableS2.docx]

Table S2. List of annotated genes without detected expression in any of the oxygen changed conditions. (.xlsx, 13 KB)

<http://www.g3journal.org/lookup/suppl/doi:10.1534/g3.116.036855/-/DC1/TableS2.xlsx>
