# Supplementary material for: The Complex Transcriptional Response of Acaryochloris marina to Different Oxygen Levels
Source: G3 (Bethesda). 2016 Dec 14;7(2):517–32. doi: 10.1534/g3.116.036855 (PMC5295598; doi:10.1534/g3.116.036855)
Supplement: Supplementary file 7 [file 517TableS3.docx]

Table S3. Genes differentially expressed. List of expressed genes (more than 50 reads) with a Log_2_FC (fold change) > 1. (.xlsx, 412 KB)

[http://www.g3journal.org/lookup/suppl/doi:10.1534/g3.116.036855/-/DC1/TableS3.xlsx](http://www.g3journal.org/lookup/suppl/doi:10.1534/g3.116.036855/-/DC1/TableS2.xlsx)
