# Supplementary material for: The Complex Transcriptional Response of Acaryochloris marina to Different Oxygen Levels
Source: G3 (Bethesda). 2016 Dec 14;7(2):517–32. doi: 10.1534/g3.116.036855 (PMC5295598; doi:10.1534/g3.116.036855)
Supplement: Supplementary file 9 [file 517TableS4.docx]

Table S4. Results of statistical analysis performed to evaluate the functional enrichment of genes differentially expressed under changed oxygen levels. (.xlsx, 13 KB)

<http://www.g3journal.org/lookup/suppl/doi:10.1534/g3.116.036855/-/DC1/TableS4.xlsx>
