# Supplementary material for: The Complex Transcriptional Response of Acaryochloris marina to Different Oxygen Levels
Source: G3 (Bethesda). 2016 Dec 14;7(2):517–32. doi: 10.1534/g3.116.036855 (PMC5295598; doi:10.1534/g3.116.036855)
Supplement: Supplementary file 11 [file 517TableS5.docx]

Table S5. Predicted targets for the ncRNAs with significant opposing regulation for microoxic and hyperoxic conditions compared to the control, i.e. AM1_NC12, AM1_NC161, AM1_NC254, AM1_NC256, AM1_NC270, and AM1_NC315. (.xlsx, 33 KB)

<http://www.g3journal.org/lookup/suppl/doi:10.1534/g3.116.036855/-/DC1/TableS5.xlsx>
