# Supplementary material for: The Complex Transcriptional Response of Acaryochloris marina to Different Oxygen Levels
Source: G3 (Bethesda). 2016 Dec 14;7(2):517–32. doi: 10.1534/g3.116.036855 (PMC5295598; doi:10.1534/g3.116.036855)
Supplement: Supplementary file 13 [file 517FileS1.docx]

**Additional data files and supplementary materials**

# The complex transcriptional response of *Acaryochloris marina* to different oxygen levels

Miguel A. Hernández-Prieto*; Yuankui Lin*; Min Chen

School of Life and Environmental Sciences, University of Sydney, NSW 2006, Australia

*These authors contributed equally to this work

**Contents:**

1. Report-summary from Rockhopper 2

2. Figure S1 (Expression of genes encoding TOs) 32

3. Figure S2 (UTRs – mRNAs correlation) 33

4. Supplemental tables

Table S1 33

Table S2 33

Table S3 34

Table S4 34

Table S5 34

# Report-summary from Rockhopper

## Alignment of control sample (replicate 1)

FATSQ files (paired end):

C_L1_1.fq C_L1_2.fq

Total reads: 3394911

Successfully aligned reads: 1805769 53% (Acaryochloris marina MBIC11017 chromosome)

Aligned (sense) to protein-coding genes: 11%

Aligned (antisense) to protein-coding genes: 10%

Aligned (sense) to ribosomal RNAs: 1%

Aligned (antisense) to ribosomal RNAs: 1%

Aligned (sense) to transfer RNAs: 0%

Aligned (antisense) to transfer RNAs: 0%

Aligned (sense) to miscellaneous RNAs: 18%

Aligned (antisense) to miscellaneous RNAs: 18%

Aligned to unannotated regions: 41%

Successfully aligned reads: 5512 0% (Acaryochloris marina MBIC11017 plasmid pREB1)

Aligned (sense) to protein-coding genes: 67%

Aligned (antisense) to protein-coding genes: 32%

Aligned (sense) to ribosomal RNAs: 0%

Aligned (antisense) to ribosomal RNAs: 0%

Aligned (sense) to transfer RNAs: 0%

Aligned (antisense) to transfer RNAs: 0%

Aligned (sense) to miscellaneous RNAs: 0%

Aligned (antisense) to miscellaneous RNAs: 0%

Aligned to unannotated regions: 1%

Successfully aligned reads: 10967 0% (Acaryochloris marina MBIC11017 plasmid pREB2)

Aligned (sense) to protein-coding genes: 26%

Aligned (antisense) to protein-coding genes: 23%

Aligned (sense) to ribosomal RNAs: 0%

Aligned (antisense) to ribosomal RNAs: 0%

Aligned (sense) to transfer RNAs: 0%

Aligned (antisense) to transfer RNAs: 0%

Aligned (sense) to miscellaneous RNAs: 0%

Aligned (antisense) to miscellaneous RNAs: 0%

Aligned to unannotated regions: 51%

Successfully aligned reads: 59313 2% (Acaryochloris marina MBIC11017 plasmid pREB3)

Aligned (sense) to protein-coding genes: 67%

Aligned (antisense) to protein-coding genes: 33%

Aligned (sense) to ribosomal RNAs: 0%

Aligned (antisense) to ribosomal RNAs: 0%

Aligned (sense) to transfer RNAs: 0%

Aligned (antisense) to transfer RNAs: 0%

Aligned (sense) to miscellaneous RNAs: 0%

Aligned (antisense) to miscellaneous RNAs: 0%

Aligned to unannotated regions: 0%

Successfully aligned reads: 3816 0% (Acaryochloris marina MBIC11017 plasmid pREB4)

Aligned (sense) to protein-coding genes: 36%

Aligned (antisense) to protein-coding genes: 34%

Aligned (sense) to ribosomal RNAs: 0%

Aligned (antisense) to ribosomal RNAs: 0%

Aligned (sense) to transfer RNAs: 0%

Aligned (antisense) to transfer RNAs: 0%

Aligned (sense) to miscellaneous RNAs: 0%

Aligned (antisense) to miscellaneous RNAs: 0%

Aligned to unannotated regions: 29%

Successfully aligned reads: 6491 0% (Acaryochloris marina MBIC11017 plasmid pREB5)

Aligned (sense) to protein-coding genes: 63%

Aligned (antisense) to protein-coding genes: 37%

Aligned (sense) to ribosomal RNAs: 0%

Aligned (antisense) to ribosomal RNAs: 0%

Aligned (sense) to transfer RNAs: 0%

Aligned (antisense) to transfer RNAs: 0%

Aligned (sense) to miscellaneous RNAs: 0%

Aligned (antisense) to miscellaneous RNAs: 0%

Aligned to unannotated regions: 0%

Successfully aligned reads: 2134 0% (Acaryochloris marina MBIC11017 plasmid pREB6)

Aligned (sense) to protein-coding genes: 35%

Aligned (antisense) to protein-coding genes: 32%

Aligned (sense) to ribosomal RNAs: 0%

Aligned (antisense) to ribosomal RNAs: 0%

Aligned (sense) to transfer RNAs: 0%

Aligned (antisense) to transfer RNAs: 0%

Aligned (sense) to miscellaneous RNAs: 0%

Aligned (antisense) to miscellaneous RNAs: 0%

Aligned to unannotated regions: 33%

Successfully aligned reads: 14641 0% (Acaryochloris marina MBIC11017 plasmid pREB7)

Aligned (sense) to protein-coding genes: 52%

Aligned (antisense) to protein-coding genes: 48%

Aligned (sense) to ribosomal RNAs: 0%

Aligned (antisense) to ribosomal RNAs: 0%

Aligned (sense) to transfer RNAs: 0%

Aligned (antisense) to transfer RNAs: 0%

Aligned (sense) to miscellaneous RNAs: 0%

Aligned (antisense) to miscellaneous RNAs: 0%

Aligned to unannotated regions: 0%

Successfully aligned reads: 1900 0% (Acaryochloris marina MBIC11017 plasmid pREB8)

Aligned (sense) to protein-coding genes: 36%

Aligned (antisense) to protein-coding genes: 35%

Aligned (sense) to ribosomal RNAs: 0%

Aligned (antisense) to ribosomal RNAs: 0%

Aligned (sense) to transfer RNAs: 0%

Aligned (antisense) to transfer RNAs: 0%

Aligned (sense) to miscellaneous RNAs: 0%

Aligned (antisense) to miscellaneous RNAs: 0%

Aligned to unannotated regions: 29%

Successfully aligned reads: 91 0% (Acaryochloris marina MBIC11017 plasmid pREB9)

Aligned (sense) to protein-coding genes: 50%

Aligned (antisense) to protein-coding genes: 38%

Aligned (sense) to ribosomal RNAs: 0%

Aligned (antisense) to ribosomal RNAs: 0%

Aligned (sense) to transfer RNAs: 0%

Aligned (antisense) to transfer RNAs: 0%

Aligned (sense) to miscellaneous RNAs: 0%

Aligned (antisense) to miscellaneous RNAs: 0%

Aligned to unannotated regions: 12%

## Alignment of control sample (replicate 2)

FATSQ files (paired end):

C2_L1_1.fq C2_L1_2.fq

Total reads: 3453356

Successfully aligned reads: 937467 27% (Acaryochloris marina MBIC11017 chromosome)

Aligned (sense) to protein-coding genes: 4%

Aligned (antisense) to protein-coding genes: 4%

Aligned (sense) to ribosomal RNAs: 0%

Aligned (antisense) to ribosomal RNAs: 0%

Aligned (sense) to transfer RNAs: 0%

Aligned (antisense) to transfer RNAs: 0%

Aligned (sense) to miscellaneous RNAs: 25%

Aligned (antisense) to miscellaneous RNAs: 24%

Aligned to unannotated regions: 42%

Successfully aligned reads: 2263 0% (Acaryochloris marina MBIC11017 plasmid pREB1)

Aligned (sense) to protein-coding genes: 65%

Aligned (antisense) to protein-coding genes: 35%

Aligned (sense) to ribosomal RNAs: 0%

Aligned (antisense) to ribosomal RNAs: 0%

Aligned (sense) to transfer RNAs: 0%

Aligned (antisense) to transfer RNAs: 0%

Aligned (sense) to miscellaneous RNAs: 0%

Aligned (antisense) to miscellaneous RNAs: 0%

Aligned to unannotated regions: 0%

Successfully aligned reads: 2910 0% (Acaryochloris marina MBIC11017 plasmid pREB2)

Aligned (sense) to protein-coding genes: 35%

Aligned (antisense) to protein-coding genes: 29%

Aligned (sense) to ribosomal RNAs: 0%

Aligned (antisense) to ribosomal RNAs: 0%

Aligned (sense) to transfer RNAs: 0%

Aligned (antisense) to transfer RNAs: 0%

Aligned (sense) to miscellaneous RNAs: 0%

Aligned (antisense) to miscellaneous RNAs: 0%

Aligned to unannotated regions: 36%

Successfully aligned reads: 3494 0% (Acaryochloris marina MBIC11017 plasmid pREB3)

Aligned (sense) to protein-coding genes: 65%

Aligned (antisense) to protein-coding genes: 35%

Aligned (sense) to ribosomal RNAs: 0%

Aligned (antisense) to ribosomal RNAs: 0%

Aligned (sense) to transfer RNAs: 0%

Aligned (antisense) to transfer RNAs: 0%

Aligned (sense) to miscellaneous RNAs: 0%

Aligned (antisense) to miscellaneous RNAs: 0%

Aligned to unannotated regions: 0%

Successfully aligned reads: 1099 0% (Acaryochloris marina MBIC11017 plasmid pREB4)

Aligned (sense) to protein-coding genes: 41%

Aligned (antisense) to protein-coding genes: 36%

Aligned (sense) to ribosomal RNAs: 0%

Aligned (antisense) to ribosomal RNAs: 0%

Aligned (sense) to transfer RNAs: 0%

Aligned (antisense) to transfer RNAs: 0%

Aligned (sense) to miscellaneous RNAs: 0%

Aligned (antisense) to miscellaneous RNAs: 0%

Aligned to unannotated regions: 23%

Successfully aligned reads: 445 0% (Acaryochloris marina MBIC11017 plasmid pREB5)

Aligned (sense) to protein-coding genes: 64%

Aligned (antisense) to protein-coding genes: 35%

Aligned (sense) to ribosomal RNAs: 0%

Aligned (antisense) to ribosomal RNAs: 0%

Aligned (sense) to transfer RNAs: 0%

Aligned (antisense) to transfer RNAs: 0%

Aligned (sense) to miscellaneous RNAs: 0%

Aligned (antisense) to miscellaneous RNAs: 0%

Aligned to unannotated regions: 0%

Successfully aligned reads: 638 0% (Acaryochloris marina MBIC11017 plasmid pREB6)

Aligned (sense) to protein-coding genes: 35%

Aligned (antisense) to protein-coding genes: 30%

Aligned (sense) to ribosomal RNAs: 0%

Aligned (antisense) to ribosomal RNAs: 0%

Aligned (sense) to transfer RNAs: 0%

Aligned (antisense) to transfer RNAs: 0%

Aligned (sense) to miscellaneous RNAs: 0%

Aligned (antisense) to miscellaneous RNAs: 0%

Aligned to unannotated regions: 35%

Successfully aligned reads: 474 0% (Acaryochloris marina MBIC11017 plasmid pREB7)

Aligned (sense) to protein-coding genes: 64%

Aligned (antisense) to protein-coding genes: 33%

Aligned (sense) to ribosomal RNAs: 0%

Aligned (antisense) to ribosomal RNAs: 0%

Aligned (sense) to transfer RNAs: 0%

Aligned (antisense) to transfer RNAs: 0%

Aligned (sense) to miscellaneous RNAs: 0%

Aligned (antisense) to miscellaneous RNAs: 0%

Aligned to unannotated regions: 3%

Successfully aligned reads: 209 0% (Acaryochloris marina MBIC11017 plasmid pREB8)

Aligned (sense) to protein-coding genes: 35%

Aligned (antisense) to protein-coding genes: 32%

Aligned (sense) to ribosomal RNAs: 0%

Aligned (antisense) to ribosomal RNAs: 0%

Aligned (sense) to transfer RNAs: 0%

Aligned (antisense) to transfer RNAs: 0%

Aligned (sense) to miscellaneous RNAs: 0%

Aligned (antisense) to miscellaneous RNAs: 0%

Aligned to unannotated regions: 33%

Successfully aligned reads: 14 0% (Acaryochloris marina MBIC11017 plasmid pREB9)

Aligned (sense) to protein-coding genes: 34%

Aligned (antisense) to protein-coding genes: 58%

Aligned (sense) to ribosomal RNAs: 0%

Aligned (antisense) to ribosomal RNAs: 0%

Aligned (sense) to transfer RNAs: 0%

Aligned (antisense) to transfer RNAs: 0%

Aligned (sense) to miscellaneous RNAs: 0%

Aligned (antisense) to miscellaneous RNAs: 0%

Aligned to unannotated regions: 8%

## Alignment of control sample (replicate 3)

FATSQ files (paired end):

C3_L1_1.fq C3_L1_2.fq

Total reads: 3369335

Successfully aligned reads: 1188950 35% (Acaryochloris marina MBIC11017 chromosome)

Aligned (sense) to protein-coding genes: 24%

Aligned (antisense) to protein-coding genes: 23%

Aligned (sense) to ribosomal RNAs: 0%

Aligned (antisense) to ribosomal RNAs: 0%

Aligned (sense) to transfer RNAs: 0%

Aligned (antisense) to transfer RNAs: 0%

Aligned (sense) to miscellaneous RNAs: 6%

Aligned (antisense) to miscellaneous RNAs: 7%

Aligned to unannotated regions: 40%

Successfully aligned reads: 13008 0% (Acaryochloris marina MBIC11017 plasmid pREB1)

Aligned (sense) to protein-coding genes: 66%

Aligned (antisense) to protein-coding genes: 34%

Aligned (sense) to ribosomal RNAs: 0%

Aligned (antisense) to ribosomal RNAs: 0%

Aligned (sense) to transfer RNAs: 0%

Aligned (antisense) to transfer RNAs: 0%

Aligned (sense) to miscellaneous RNAs: 0%

Aligned (antisense) to miscellaneous RNAs: 0%

Aligned to unannotated regions: 0%

Successfully aligned reads: 17217 1% (Acaryochloris marina MBIC11017 plasmid pREB2)

Aligned (sense) to protein-coding genes: 30%

Aligned (antisense) to protein-coding genes: 29%

Aligned (sense) to ribosomal RNAs: 0%

Aligned (antisense) to ribosomal RNAs: 0%

Aligned (sense) to transfer RNAs: 0%

Aligned (antisense) to transfer RNAs: 0%

Aligned (sense) to miscellaneous RNAs: 0%

Aligned (antisense) to miscellaneous RNAs: 0%

Aligned to unannotated regions: 41%

Successfully aligned reads: 172000 5% (Acaryochloris marina MBIC11017 plasmid pREB3)

Aligned (sense) to protein-coding genes: 65%

Aligned (antisense) to protein-coding genes: 35%

Aligned (sense) to ribosomal RNAs: 0%

Aligned (antisense) to ribosomal RNAs: 0%

Aligned (sense) to transfer RNAs: 0%

Aligned (antisense) to transfer RNAs: 0%

Aligned (sense) to miscellaneous RNAs: 0%

Aligned (antisense) to miscellaneous RNAs: 0%

Aligned to unannotated regions: 0%

Successfully aligned reads: 5091 0% (Acaryochloris marina MBIC11017 plasmid pREB4)

Aligned (sense) to protein-coding genes: 39%

Aligned (antisense) to protein-coding genes: 37%

Aligned (sense) to ribosomal RNAs: 0%

Aligned (antisense) to ribosomal RNAs: 0%

Aligned (sense) to transfer RNAs: 0%

Aligned (antisense) to transfer RNAs: 0%

Aligned (sense) to miscellaneous RNAs: 0%

Aligned (antisense) to miscellaneous RNAs: 0%

Aligned to unannotated regions: 25%

Successfully aligned reads: 7604 0% (Acaryochloris marina MBIC11017 plasmid pREB5)

Aligned (sense) to protein-coding genes: 62%

Aligned (antisense) to protein-coding genes: 38%

Aligned (sense) to ribosomal RNAs: 0%

Aligned (antisense) to ribosomal RNAs: 0%

Aligned (sense) to transfer RNAs: 0%

Aligned (antisense) to transfer RNAs: 0%

Aligned (sense) to miscellaneous RNAs: 0%

Aligned (antisense) to miscellaneous RNAs: 0%

Aligned to unannotated regions: 0%

Successfully aligned reads: 3921 0% (Acaryochloris marina MBIC11017 plasmid pREB6)

Aligned (sense) to protein-coding genes: 34%

Aligned (antisense) to protein-coding genes: 30%

Aligned (sense) to ribosomal RNAs: 0%

Aligned (antisense) to ribosomal RNAs: 0%

Aligned (sense) to transfer RNAs: 0%

Aligned (antisense) to transfer RNAs: 0%

Aligned (sense) to miscellaneous RNAs: 0%

Aligned (antisense) to miscellaneous RNAs: 0%

Aligned to unannotated regions: 36%

Successfully aligned reads: 10565 0% (Acaryochloris marina MBIC11017 plasmid pREB7)

Aligned (sense) to protein-coding genes: 58%

Aligned (antisense) to protein-coding genes: 41%

Aligned (sense) to ribosomal RNAs: 0%

Aligned (antisense) to ribosomal RNAs: 0%

Aligned (sense) to transfer RNAs: 0%

Aligned (antisense) to transfer RNAs: 0%

Aligned (sense) to miscellaneous RNAs: 0%

Aligned (antisense) to miscellaneous RNAs: 0%

Aligned to unannotated regions: 1%

Successfully aligned reads: 2965 0% (Acaryochloris marina MBIC11017 plasmid pREB8)

Aligned (sense) to protein-coding genes: 38%

Aligned (antisense) to protein-coding genes: 35%

Aligned (sense) to ribosomal RNAs: 0%

Aligned (antisense) to ribosomal RNAs: 0%

Aligned (sense) to transfer RNAs: 0%

Aligned (antisense) to transfer RNAs: 0%

Aligned (sense) to miscellaneous RNAs: 0%

Aligned (antisense) to miscellaneous RNAs: 0%

Aligned to unannotated regions: 27%

Successfully aligned reads: 44 0% (Acaryochloris marina MBIC11017 plasmid pREB9)

Aligned (sense) to protein-coding genes: 48%

Aligned (antisense) to protein-coding genes: 41%

Aligned (sense) to ribosomal RNAs: 0%

Aligned (antisense) to ribosomal RNAs: 0%

Aligned (sense) to transfer RNAs: 0%

Aligned (antisense) to transfer RNAs: 0%

Aligned (sense) to miscellaneous RNAs: 0%

Aligned (antisense) to miscellaneous RNAs: 0%

Aligned to unannotated regions: 11%

## Alignment of microoxic sample (replicate 1)

FATSQ files (paired end):

Aligned sequencing reads from files:

N1_L1_1.fq N1_L1_2.fq

Total reads: 3287764

Successfully aligned reads: 1647825 50% (Acaryochloris marina MBIC11017 chromosome)

Aligned (sense) to protein-coding genes: 29%

Aligned (antisense) to protein-coding genes: 29%

Aligned (sense) to ribosomal RNAs: 1%

Aligned (antisense) to ribosomal RNAs: 1%

Aligned (sense) to transfer RNAs: 0%

Aligned (antisense) to transfer RNAs: 0%

Aligned (sense) to miscellaneous RNAs: 4%

Aligned (antisense) to miscellaneous RNAs: 4%

Aligned to unannotated regions: 32%

Successfully aligned reads: 23079 1% (Acaryochloris marina MBIC11017 plasmid pREB1)

Aligned (sense) to protein-coding genes: 69%

Aligned (antisense) to protein-coding genes: 30%

Aligned (sense) to ribosomal RNAs: 0%

Aligned (antisense) to ribosomal RNAs: 0%

Aligned (sense) to transfer RNAs: 0%

Aligned (antisense) to transfer RNAs: 0%

Aligned (sense) to miscellaneous RNAs: 0%

Aligned (antisense) to miscellaneous RNAs: 0%

Aligned to unannotated regions: 0%

Successfully aligned reads: 49887 2% (Acaryochloris marina MBIC11017 plasmid pREB2)

Aligned (sense) to protein-coding genes: 31%

Aligned (antisense) to protein-coding genes: 30%

Aligned (sense) to ribosomal RNAs: 0%

Aligned (antisense) to ribosomal RNAs: 0%

Aligned (sense) to transfer RNAs: 0%

Aligned (antisense) to transfer RNAs: 0%

Aligned (sense) to miscellaneous RNAs: 0%

Aligned (antisense) to miscellaneous RNAs: 0%

Aligned to unannotated regions: 39%

Successfully aligned reads: 89808 3% (Acaryochloris marina MBIC11017 plasmid pREB3)

Aligned (sense) to protein-coding genes: 64%

Aligned (antisense) to protein-coding genes: 36%

Aligned (sense) to ribosomal RNAs: 0%

Aligned (antisense) to ribosomal RNAs: 0%

Aligned (sense) to transfer RNAs: 0%

Aligned (antisense) to transfer RNAs: 0%

Aligned (sense) to miscellaneous RNAs: 0%

Aligned (antisense) to miscellaneous RNAs: 0%

Aligned to unannotated regions: 0%

Successfully aligned reads: 17411 1% (Acaryochloris marina MBIC11017 plasmid pREB4)

Aligned (sense) to protein-coding genes: 35%

Aligned (antisense) to protein-coding genes: 34%

Aligned (sense) to ribosomal RNAs: 0%

Aligned (antisense) to ribosomal RNAs: 0%

Aligned (sense) to transfer RNAs: 0%

Aligned (antisense) to transfer RNAs: 0%

Aligned (sense) to miscellaneous RNAs: 0%

Aligned (antisense) to miscellaneous RNAs: 0%

Aligned to unannotated regions: 32%

Successfully aligned reads: 26628 1% (Acaryochloris marina MBIC11017 plasmid pREB5)

Aligned (sense) to protein-coding genes: 61%

Aligned (antisense) to protein-coding genes: 39%

Aligned (sense) to ribosomal RNAs: 0%

Aligned (antisense) to ribosomal RNAs: 0%

Aligned (sense) to transfer RNAs: 0%

Aligned (antisense) to transfer RNAs: 0%

Aligned (sense) to miscellaneous RNAs: 0%

Aligned (antisense) to miscellaneous RNAs: 0%

Aligned to unannotated regions: 0%

Successfully aligned reads: 10055 0% (Acaryochloris marina MBIC11017 plasmid pREB6)

Aligned (sense) to protein-coding genes: 35%

Aligned (antisense) to protein-coding genes: 33%

Aligned (sense) to ribosomal RNAs: 0%

Aligned (antisense) to ribosomal RNAs: 0%

Aligned (sense) to transfer RNAs: 0%

Aligned (antisense) to transfer RNAs: 0%

Aligned (sense) to miscellaneous RNAs: 0%

Aligned (antisense) to miscellaneous RNAs: 0%

Aligned to unannotated regions: 31%

Successfully aligned reads: 20812 1% (Acaryochloris marina MBIC11017 plasmid pREB7)

Aligned (sense) to protein-coding genes: 61%

Aligned (antisense) to protein-coding genes: 37%

Aligned (sense) to ribosomal RNAs: 0%

Aligned (antisense) to ribosomal RNAs: 0%

Aligned (sense) to transfer RNAs: 0%

Aligned (antisense) to transfer RNAs: 0%

Aligned (sense) to miscellaneous RNAs: 0%

Aligned (antisense) to miscellaneous RNAs: 0%

Aligned to unannotated regions: 1%

Successfully aligned reads: 14987 0% (Acaryochloris marina MBIC11017 plasmid pREB8)

Aligned (sense) to protein-coding genes: 32%

Aligned (antisense) to protein-coding genes: 31%

Aligned (sense) to ribosomal RNAs: 0%

Aligned (antisense) to ribosomal RNAs: 0%

Aligned (sense) to transfer RNAs: 0%

Aligned (antisense) to transfer RNAs: 0%

Aligned (sense) to miscellaneous RNAs: 0%

Aligned (antisense) to miscellaneous RNAs: 0%

Aligned to unannotated regions: 37%

Successfully aligned reads: 99 0% (Acaryochloris marina MBIC11017 plasmid pREB9)

Aligned (sense) to protein-coding genes: 49%

Aligned (antisense) to protein-coding genes: 47%

Aligned (sense) to ribosomal RNAs: 0%

Aligned (antisense) to ribosomal RNAs: 0%

Aligned (sense) to transfer RNAs: 0%

Aligned (antisense) to transfer RNAs: 0%

Aligned (sense) to miscellaneous RNAs: 0%

Aligned (antisense) to miscellaneous RNAs: 0%

Aligned to unannotated regions: 5%

## Alignment of microoxic (replicate 2)

Aligned sequencing reads from files:

N2_L1_1.fq N2_L1_2.fq

Total reads: 3306436

Successfully aligned reads: 1514030 46% (Acaryochloris marina MBIC11017 chromosome)

Aligned (sense) to protein-coding genes: 22%

Aligned (antisense) to protein-coding genes: 21%

Aligned (sense) to ribosomal RNAs: 0%

Aligned (antisense) to ribosomal RNAs: 0%

Aligned (sense) to transfer RNAs: 0%

Aligned (antisense) to transfer RNAs: 0%

Aligned (sense) to miscellaneous RNAs: 14%

Aligned (antisense) to miscellaneous RNAs: 14%

Aligned to unannotated regions: 28%

Successfully aligned reads: 21983 1% (Acaryochloris marina MBIC11017 plasmid pREB1)

Aligned (sense) to protein-coding genes: 71%

Aligned (antisense) to protein-coding genes: 29%

Aligned (sense) to ribosomal RNAs: 0%

Aligned (antisense) to ribosomal RNAs: 0%

Aligned (sense) to transfer RNAs: 0%

Aligned (antisense) to transfer RNAs: 0%

Aligned (sense) to miscellaneous RNAs: 0%

Aligned (antisense) to miscellaneous RNAs: 0%

Aligned to unannotated regions: 0%

Successfully aligned reads: 29926 1% (Acaryochloris marina MBIC11017 plasmid pREB2)

Aligned (sense) to protein-coding genes: 38%

Aligned (antisense) to protein-coding genes: 37%

Aligned (sense) to ribosomal RNAs: 0%

Aligned (antisense) to ribosomal RNAs: 0%

Aligned (sense) to transfer RNAs: 0%

Aligned (antisense) to transfer RNAs: 0%

Aligned (sense) to miscellaneous RNAs: 0%

Aligned (antisense) to miscellaneous RNAs: 0%

Aligned to unannotated regions: 25%

Successfully aligned reads: 104161 3% (Acaryochloris marina MBIC11017 plasmid pREB3)

Aligned (sense) to protein-coding genes: 66%

Aligned (antisense) to protein-coding genes: 34%

Aligned (sense) to ribosomal RNAs: 0%

Aligned (antisense) to ribosomal RNAs: 0%

Aligned (sense) to transfer RNAs: 0%

Aligned (antisense) to transfer RNAs: 0%

Aligned (sense) to miscellaneous RNAs: 0%

Aligned (antisense) to miscellaneous RNAs: 0%

Aligned to unannotated regions: 0%

Successfully aligned reads: 9538 0% (Acaryochloris marina MBIC11017 plasmid pREB4)

Aligned (sense) to protein-coding genes: 41%

Aligned (antisense) to protein-coding genes: 41%

Aligned (sense) to ribosomal RNAs: 0%

Aligned (antisense) to ribosomal RNAs: 0%

Aligned (sense) to transfer RNAs: 0%

Aligned (antisense) to transfer RNAs: 0%

Aligned (sense) to miscellaneous RNAs: 0%

Aligned (antisense) to miscellaneous RNAs: 0%

Aligned to unannotated regions: 18%

Successfully aligned reads: 6876 0% (Acaryochloris marina MBIC11017 plasmid pREB5)

Aligned (sense) to protein-coding genes: 70%

Aligned (antisense) to protein-coding genes: 30%

Aligned (sense) to ribosomal RNAs: 0%

Aligned (antisense) to ribosomal RNAs: 0%

Aligned (sense) to transfer RNAs: 0%

Aligned (antisense) to transfer RNAs: 0%

Aligned (sense) to miscellaneous RNAs: 0%

Aligned (antisense) to miscellaneous RNAs: 0%

Aligned to unannotated regions: 0%

Successfully aligned reads: 7425 0% (Acaryochloris marina MBIC11017 plasmid pREB6)

Aligned (sense) to protein-coding genes: 36%

Aligned (antisense) to protein-coding genes: 35%

Aligned (sense) to ribosomal RNAs: 0%

Aligned (antisense) to ribosomal RNAs: 0%

Aligned (sense) to transfer RNAs: 0%

Aligned (antisense) to transfer RNAs: 0%

Aligned (sense) to miscellaneous RNAs: 0%

Aligned (antisense) to miscellaneous RNAs: 0%

Aligned to unannotated regions: 29%

Successfully aligned reads: 6419 0% (Acaryochloris marina MBIC11017 plasmid pREB7)

Aligned (sense) to protein-coding genes: 66%

Aligned (antisense) to protein-coding genes: 30%

Aligned (sense) to ribosomal RNAs: 0%

Aligned (antisense) to ribosomal RNAs: 0%

Aligned (sense) to transfer RNAs: 0%

Aligned (antisense) to transfer RNAs: 0%

Aligned (sense) to miscellaneous RNAs: 0%

Aligned (antisense) to miscellaneous RNAs: 0%

Aligned to unannotated regions: 4%

Successfully aligned reads: 4783 0% (Acaryochloris marina MBIC11017 plasmid pREB8)

Aligned (sense) to protein-coding genes: 40%

Aligned (antisense) to protein-coding genes: 40%

Aligned (sense) to ribosomal RNAs: 0%

Aligned (antisense) to ribosomal RNAs: 0%

Aligned (sense) to transfer RNAs: 0%

Aligned (antisense) to transfer RNAs: 0%

Aligned (sense) to miscellaneous RNAs: 0%

Aligned (antisense) to miscellaneous RNAs: 0%

Aligned to unannotated regions: 20%

Successfully aligned reads: 48 0% (Acaryochloris marina MBIC11017 plasmid pREB9)

Aligned (sense) to protein-coding genes: 51%

Aligned (antisense) to protein-coding genes: 46%

Aligned (sense) to ribosomal RNAs: 0%

Aligned (antisense) to ribosomal RNAs: 0%

Aligned (sense) to transfer RNAs: 0%

Aligned (antisense) to transfer RNAs: 0%

Aligned (sense) to miscellaneous RNAs: 0%

Aligned (antisense) to miscellaneous RNAs: 0%

Aligned to unannotated regions: 4%

## Alignment of hyperoxic (replicate 1)

Aligned sequencing reads from files:

O1_L1_1.fq O1_L1_2.fq

Total reads: 3397332

Successfully aligned reads: 2918916 86% (Acaryochloris marina MBIC11017 chromosome)

Aligned (sense) to protein-coding genes: 10%

Aligned (antisense) to protein-coding genes: 10%

Aligned (sense) to ribosomal RNAs: 2%

Aligned (antisense) to ribosomal RNAs: 1%

Aligned (sense) to transfer RNAs: 0%

Aligned (antisense) to transfer RNAs: 0%

Aligned (sense) to miscellaneous RNAs: 18%

Aligned (antisense) to miscellaneous RNAs: 18%

Aligned to unannotated regions: 41%

Successfully aligned reads: 8231 0% (Acaryochloris marina MBIC11017 plasmid pREB1)

Aligned (sense) to protein-coding genes: 68%

Aligned (antisense) to protein-coding genes: 32%

Aligned (sense) to ribosomal RNAs: 0%

Aligned (antisense) to ribosomal RNAs: 0%

Aligned (sense) to transfer RNAs: 0%

Aligned (antisense) to transfer RNAs: 0%

Aligned (sense) to miscellaneous RNAs: 0%

Aligned (antisense) to miscellaneous RNAs: 0%

Aligned to unannotated regions: 0%

Successfully aligned reads: 13344 0% (Acaryochloris marina MBIC11017 plasmid pREB2)

Aligned (sense) to protein-coding genes: 29%

Aligned (antisense) to protein-coding genes: 27%

Aligned (sense) to ribosomal RNAs: 0%

Aligned (antisense) to ribosomal RNAs: 0%

Aligned (sense) to transfer RNAs: 0%

Aligned (antisense) to transfer RNAs: 0%

Aligned (sense) to miscellaneous RNAs: 0%

Aligned (antisense) to miscellaneous RNAs: 0%

Aligned to unannotated regions: 44%

Successfully aligned reads: 54028 2% (Acaryochloris marina MBIC11017 plasmid pREB3)

Aligned (sense) to protein-coding genes: 63%

Aligned (antisense) to protein-coding genes: 37%

Aligned (sense) to ribosomal RNAs: 0%

Aligned (antisense) to ribosomal RNAs: 0%

Aligned (sense) to transfer RNAs: 0%

Aligned (antisense) to transfer RNAs: 0%

Aligned (sense) to miscellaneous RNAs: 0%

Aligned (antisense) to miscellaneous RNAs: 0%

Aligned to unannotated regions: 0%

Successfully aligned reads: 4252 0% (Acaryochloris marina MBIC11017 plasmid pREB4)

Aligned (sense) to protein-coding genes: 37%

Aligned (antisense) to protein-coding genes: 35%

Aligned (sense) to ribosomal RNAs: 0%

Aligned (antisense) to ribosomal RNAs: 0%

Aligned (sense) to transfer RNAs: 0%

Aligned (antisense) to transfer RNAs: 0%

Aligned (sense) to miscellaneous RNAs: 0%

Aligned (antisense) to miscellaneous RNAs: 0%

Aligned to unannotated regions: 28%

Successfully aligned reads: 4498 0% (Acaryochloris marina MBIC11017 plasmid pREB5)

Aligned (sense) to protein-coding genes: 65%

Aligned (antisense) to protein-coding genes: 35%

Aligned (sense) to ribosomal RNAs: 0%

Aligned (antisense) to ribosomal RNAs: 0%

Aligned (sense) to transfer RNAs: 0%

Aligned (antisense) to transfer RNAs: 0%

Aligned (sense) to miscellaneous RNAs: 0%

Aligned (antisense) to miscellaneous RNAs: 0%

Aligned to unannotated regions: 0%

Successfully aligned reads: 11646 0% (Acaryochloris marina MBIC11017 plasmid pREB6)

Aligned (sense) to protein-coding genes: 38%

Aligned (antisense) to protein-coding genes: 36%

Aligned (sense) to ribosomal RNAs: 0%

Aligned (antisense) to ribosomal RNAs: 0%

Aligned (sense) to transfer RNAs: 0%

Aligned (antisense) to transfer RNAs: 0%

Aligned (sense) to miscellaneous RNAs: 0%

Aligned (antisense) to miscellaneous RNAs: 0%

Aligned to unannotated regions: 27%

Successfully aligned reads: 6926 0% (Acaryochloris marina MBIC11017 plasmid pREB7)

Aligned (sense) to protein-coding genes: 62%

Aligned (antisense) to protein-coding genes: 37%

Aligned (sense) to ribosomal RNAs: 0%

Aligned (antisense) to ribosomal RNAs: 0%

Aligned (sense) to transfer RNAs: 0%

Aligned (antisense) to transfer RNAs: 0%

Aligned (sense) to miscellaneous RNAs: 0%

Aligned (antisense) to miscellaneous RNAs: 0%

Aligned to unannotated regions: 1%

Successfully aligned reads: 1983 0% (Acaryochloris marina MBIC11017 plasmid pREB8)

Aligned (sense) to protein-coding genes: 33%

Aligned (antisense) to protein-coding genes: 34%

Aligned (sense) to ribosomal RNAs: 0%

Aligned (antisense) to ribosomal RNAs: 0%

Aligned (sense) to transfer RNAs: 0%

Aligned (antisense) to transfer RNAs: 0%

Aligned (sense) to miscellaneous RNAs: 0%

Aligned (antisense) to miscellaneous RNAs: 0%

Aligned to unannotated regions: 33%

Successfully aligned reads: 70 0% (Acaryochloris marina MBIC11017 plasmid pREB9)

Aligned (sense) to protein-coding genes: 46%

Aligned (antisense) to protein-coding genes: 49%

Aligned (sense) to ribosomal RNAs: 0%

Aligned (antisense) to ribosomal RNAs: 0%

Aligned (sense) to transfer RNAs: 0%

Aligned (antisense) to transfer RNAs: 0%

Aligned (sense) to miscellaneous RNAs: 0%

Aligned (antisense) to miscellaneous RNAs: 0%

Aligned to unannotated regions: 5%

## Alignment of hyperoxic (replicate 2)

Aligned sequencing reads from files:

O2_L1_1.fq O2_L1_2.fq

Total reads: 3439935

Successfully aligned reads: 2416538 70% (Acaryochloris marina MBIC11017 chromosome)

Aligned (sense) to protein-coding genes: 21%

Aligned (antisense) to protein-coding genes: 19%

Aligned (sense) to ribosomal RNAs: 0%

Aligned (antisense) to ribosomal RNAs: 0%

Aligned (sense) to transfer RNAs: 0%

Aligned (antisense) to transfer RNAs: 0%

Aligned (sense) to miscellaneous RNAs: 10%

Aligned (antisense) to miscellaneous RNAs: 11%

Aligned to unannotated regions: 38%

Successfully aligned reads: 53276 2% (Acaryochloris marina MBIC11017 plasmid pREB1)

Aligned (sense) to protein-coding genes: 66%

Aligned (antisense) to protein-coding genes: 34%

Aligned (sense) to ribosomal RNAs: 0%

Aligned (antisense) to ribosomal RNAs: 0%

Aligned (sense) to transfer RNAs: 0%

Aligned (antisense) to transfer RNAs: 0%

Aligned (sense) to miscellaneous RNAs: 0%

Aligned (antisense) to miscellaneous RNAs: 0%

Aligned to unannotated regions: 0%

Successfully aligned reads: 52094 2% (Acaryochloris marina MBIC11017 plasmid pREB2)

Aligned (sense) to protein-coding genes: 35%

Aligned (antisense) to protein-coding genes: 34%

Aligned (sense) to ribosomal RNAs: 0%

Aligned (antisense) to ribosomal RNAs: 0%

Aligned (sense) to transfer RNAs: 0%

Aligned (antisense) to transfer RNAs: 0%

Aligned (sense) to miscellaneous RNAs: 0%

Aligned (antisense) to miscellaneous RNAs: 0%

Aligned to unannotated regions: 31%

Successfully aligned reads: 163861 5% (Acaryochloris marina MBIC11017 plasmid pREB3)

Aligned (sense) to protein-coding genes: 64%

Aligned (antisense) to protein-coding genes: 36%

Aligned (sense) to ribosomal RNAs: 0%

Aligned (antisense) to ribosomal RNAs: 0%

Aligned (sense) to transfer RNAs: 0%

Aligned (antisense) to transfer RNAs: 0%

Aligned (sense) to miscellaneous RNAs: 0%

Aligned (antisense) to miscellaneous RNAs: 0%

Aligned to unannotated regions: 0%

Successfully aligned reads: 17277 1% (Acaryochloris marina MBIC11017 plasmid pREB4)

Aligned (sense) to protein-coding genes: 38%

Aligned (antisense) to protein-coding genes: 38%

Aligned (sense) to ribosomal RNAs: 0%

Aligned (antisense) to ribosomal RNAs: 0%

Aligned (sense) to transfer RNAs: 0%

Aligned (antisense) to transfer RNAs: 0%

Aligned (sense) to miscellaneous RNAs: 0%

Aligned (antisense) to miscellaneous RNAs: 0%

Aligned to unannotated regions: 24%

Successfully aligned reads: 18991 1% (Acaryochloris marina MBIC11017 plasmid pREB5)

Aligned (sense) to protein-coding genes: 65%

Aligned (antisense) to protein-coding genes: 35%

Aligned (sense) to ribosomal RNAs: 0%

Aligned (antisense) to ribosomal RNAs: 0%

Aligned (sense) to transfer RNAs: 0%

Aligned (antisense) to transfer RNAs: 0%

Aligned (sense) to miscellaneous RNAs: 0%

Aligned (antisense) to miscellaneous RNAs: 0%

Aligned to unannotated regions: 0%

Successfully aligned reads: 16419 0% (Acaryochloris marina MBIC11017 plasmid pREB6)

Aligned (sense) to protein-coding genes: 33%

Aligned (antisense) to protein-coding genes: 31%

Aligned (sense) to ribosomal RNAs: 0%

Aligned (antisense) to ribosomal RNAs: 0%

Aligned (sense) to transfer RNAs: 0%

Aligned (antisense) to transfer RNAs: 0%

Aligned (sense) to miscellaneous RNAs: 0%

Aligned (antisense) to miscellaneous RNAs: 0%

Aligned to unannotated regions: 36%

Successfully aligned reads: 26640 1% (Acaryochloris marina MBIC11017 plasmid pREB7)

Aligned (sense) to protein-coding genes: 59%

Aligned (antisense) to protein-coding genes: 40%

Aligned (sense) to ribosomal RNAs: 0%

Aligned (antisense) to ribosomal RNAs: 0%

Aligned (sense) to transfer RNAs: 0%

Aligned (antisense) to transfer RNAs: 0%

Aligned (sense) to miscellaneous RNAs: 0%

Aligned (antisense) to miscellaneous RNAs: 0%

Aligned to unannotated regions: 1%

Successfully aligned reads: 9313 0% (Acaryochloris marina MBIC11017 plasmid pREB8)

Aligned (sense) to protein-coding genes: 36%

Aligned (antisense) to protein-coding genes: 35%

Aligned (sense) to ribosomal RNAs: 0%

Aligned (antisense) to ribosomal RNAs: 0%

Aligned (sense) to transfer RNAs: 0%

Aligned (antisense) to transfer RNAs: 0%

Aligned (sense) to miscellaneous RNAs: 0%

Aligned (antisense) to miscellaneous RNAs: 0%

Aligned to unannotated regions: 29%

Successfully aligned reads: 159 0% (Acaryochloris marina MBIC11017 plasmid pREB9)

Aligned (sense) to protein-coding genes: 48%

Aligned (antisense) to protein-coding genes: 46%

Aligned (sense) to ribosomal RNAs: 0%

Aligned (antisense) to ribosomal RNAs: 0%

Aligned (sense) to transfer RNAs: 0%

Aligned (antisense) to transfer RNAs: 0%

Aligned (sense) to miscellaneous RNAs: 0%

Aligned (antisense) to miscellaneous RNAs: 0%

Aligned to unannotated regions: 5%

## Transcripts detected after alignment

**Acaryochloris marina MBIC11017 chromosome**

Number of 5'UTRs: 2304

Number of 3'UTRs: 1910

Number of predicted RNAs: 360

Number of gene-pairs predicted to be part of the same operon: 1610

Number of predicted multi-gene operons: 1081

**Acaryochloris marina MBIC11017 plasmid pREB1**

Number of 5'UTRs: 114

Number of 3'UTRs: 105

Number of predicted RNAs: 5

Number of gene-pairs predicted to be part of the same operon: 122

Number of predicted multi-gene operons: 77

**Acaryochloris marina MBIC11017 plasmid pREB2**

Number of 5'UTRs: 110

Number of 3'UTRs: 101

Number of predicted RNAs: 14

Computing likely operons...

Number of gene-pairs predicted to be part of the same operon: 123

Number of predicted multi-gene operons: 79

**Acaryochloris marina MBIC11017 plasmid pREB3**

Number of 5'UTRs: 143

Number of 3'UTRs: 158

Number of predicted RNAs: 36

Number of gene-pairs predicted to be part of the same operon: 97

Number of predicted multi-gene operons: 71

**Acaryochloris marina MBIC11017 plasmid pREB4**

Number of 5'UTRs: 75

Number of 3'UTRs: 74

Number of predicted RNAs: 7

Number of gene-pairs predicted to be part of the same operon: 96

Number of predicted multi-gene operons: 57

**Acaryochloris marina MBIC11017 plasmid pREB5**

Number of 5'UTRs: 66

Number of 3'UTRs: 58

Number of predicted RNAs: 7

Number of gene-pairs predicted to be part of the same operon: 75

Number of predicted multi-gene operons: 44

**Acaryochloris marina MBIC11017 plasmid pREB6**

Number of 5'UTRs: 64

Number of 3'UTRs: 56

Number of predicted RNAs: 10

Number of gene-pairs predicted to be part of the same operon: 50

Number of predicted multi-gene operons: 30

**Acaryochloris marina MBIC11017 plasmid pREB7**

Number of 5'UTRs: 46

Number of 3'UTRs: 46

Number of predicted RNAs: 8

Number of gene-pairs predicted to be part of the same operon: 41

Number of predicted multi-gene operons: 30

**Acaryochloris marina MBIC11017 plasmid pREB8**

Number of 5'UTRs: 30

Number of 3'UTRs: 32

Number of predicted RNAs: 4

Number of gene-pairs predicted to be part of the same operon: 28

Number of predicted multi-gene operons: 16

**Acaryochloris marina MBIC11017 plasmid pREB9**

Number of 5'UTRs: 2

Number of 3'UTRs: 1

Number of predicted RNAs: 0

Number of gene-pairs predicted to be part of the same operon: 1

Number of predicted multi-gene operons: 1

# Figure S1


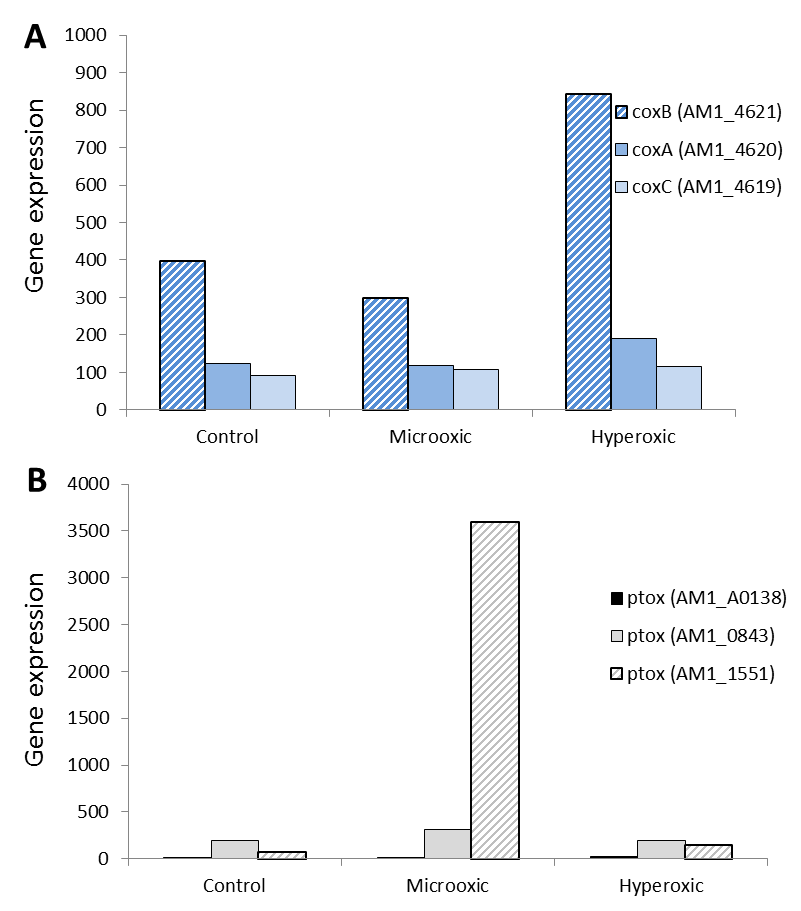


**Figure S1.** Expression levels of genes encoding terminal oxidases. The gene expression values on the Y-axis represent reads per kilobase per million mapped reads (RPKM).

# Figure S2


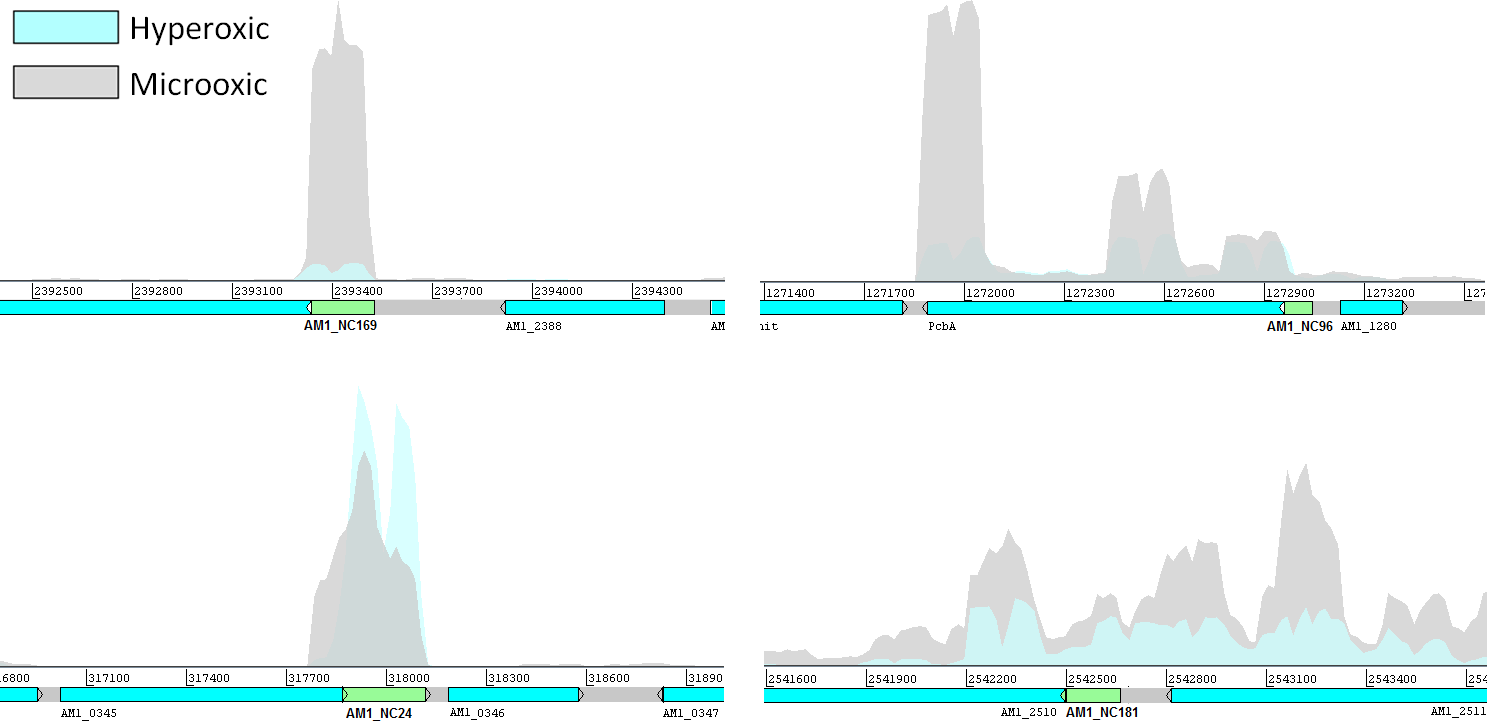


**Figure S2.** UTR regions with expression pattern not correlated with the expression pattern of the adjacent gene upon changed oxygen levels (spearman correlation, *r_s_* < 0.6).

# Supplemental tables

**Table S1.** Genes lists within functional categories and subcategories represented in CyanoBase, KEGG pathways and Gene Ontology (GO). The functional enrichment analysis of the gene clusters is based on this classification system.

[See attached file: Supplementary Table S1]

**Table S2.** List of annotated genes without detected expression in any of the oxygen changed conditions.

| **Gene ID** | **Product (Cyanobase Anotation)** | **Coordinates (translation)** | **Expression Control** | **Expression Microoxic** | **Expression Hyperoxic** |
| --- | --- | --- | --- | --- | --- |
| AM1_0242 | hypothetical protein | 240309, 240193 | 0 | 0 | 0 |
| AM1_5007 | hypothetical protein | 5054988, 5055107 | 0 | 0 | 0 |
| AM1_5292 | hypothetical protein | 5357958, 5357404 | 0 | 0 | 0 |
| AM1_5837 | hypothetical protein | 5909464, 5909348 | 0 | 0 | 0 |
| AM1_5840 | hypothetical protein | 5911211, 5911444 | 0 | 0 | 0 |
| AM1_A0163 | Iron(III) dicitrate ABC transporter, permease protein | 144789, 143812 | 0 | 0 | 0 |
| AM1_A0327 | hypothetical protein | 328016, 327756 | 0 | 0 | 0 |
| AM1_B0198 | hypothetical protein | 148110, 147943 | 0 | 0 | 0 |
| AM1_B0217 | hypothetical protein | 160175, 159798 | 0 | 0 | 0 |
| AM1_C0030 | hypothetical protein | 31792, 30344 | 0 | 0 | 0 |
| AM1_C0031 | hypothetical protein | 32511, 31792 | 0 | 0 | 0 |
| AM1_D0170 | hypothetical protein | 133545, 133426 | 0 | 0 | 0 |
| AM1_D0224 | hypothetical protein | 182930, 183073 | 0 | 0 | 0 |
| AM1_E0070 | hypothetical protein | 46322, 46182 | 0 | 0 | 0 |
| AM1_E0147 | hypothetical protein | 109954, 109652 | 0 | 0 | 0 |

**Table S3.** Genes differentially expressed. List of expressed genes (more than 50 reads) with a Log_2_FC (fold change) > 1.

[See attached file: Supplementary Table S3]

**Table S4.** Results of statistical analysis performed to evaluate the functional enrichment of genes differentially expressed under changed oxygen levels. Two different classification methods were used, KEGG pathways and GO terms. The results for each condition are in separated spread sheets.

[See attached file: Supplementary Table S4]

**Table S5.** Predicted targets for the ncRNAs with significant opposing regulation for microoxic and hyperoxic conditions compared to the control, i.e. AM1_NC12, AM1_NC161, AM1_NC254, AM1_NC256, AM1_NC270, and AM1_NC315. The results presented on the table were obtained using the IntaRNA algorithms with the exception of “Expression Correlation” which was calculated comparing the expression of the studied ncRNA with that of the predicted targets. The results for each ncRNA are in separated spread sheets.

[See attached file: Supplementary Table S5]
